# Molecular evolution of a reproductive barrier in maize and related species

**DOI:** 10.1093/genetics/iyaf085

**Published:** 2025-05-08

**Authors:** Elli Cryan, Garnet Phinney, Arun S Seetharam, Matthew M S Evans, Elizabeth A Kellogg, Junpeng Zhan, Blake Meyers, Daniel J Kliebenstein, Jeffrey Ross-Ibarra

**Affiliations:** Plant Sciences, University of California Davis, Davis, CA 95616, USA; Evolution and Ecology, University of California Davis, Davis, CA 95616, USA; Center for Population Biology, University of California Davis, Davis, CA 95616, USA; Evolution and Ecology, University of California Davis, Davis, CA 95616, USA; Ecology, Evolution and Organismal Biology, Iowa State University, Ames, IA 50011, USA; Genetics, Development and Cell Biology, Iowa State University, Ames, IA 50011, USA; Plant Biology, Carnegie Institution for Science, Stanford, CA 94305, USA; Donald Danforth Plant Science Center, St. Louis, MO 63132, USA; Arnold Arboretum of Harvard University, Boston, MA 02130, USA; Donald Danforth Plant Science Center, St. Louis, MO 63132, USA; National Key Laboratory of Crop Genetic Improvement, Huazhong Agricultural University, Wuhan, Hubei 430070, China; College of Life Science and Technology, Hubei Hongshan Laboratory, Wuhan, Hubei 430070, China; Plant Sciences, University of California Davis, Davis, CA 95616, USA; Donald Danforth Plant Science Center, St. Louis, MO 63132, USA; Plant Science and Technology, University of Missouri—Columbia, Columbia, MO 65211, USA; Genome Center, University of California Davis, Davis, CA 95616, USA; Plant Sciences, University of California Davis, Davis, CA 95616, USA; Center for Population Biology, University of California Davis, Davis, CA 95616, USA; Genome Center, University of California Davis, Davis, CA 95616, USA; Evolution and Ecology, University of California Davis, Davis, CA 95616, USA; Center for Population Biology, University of California Davis, Davis, CA 95616, USA; Genome Center, University of California Davis, Davis, CA 95616, USA

**Keywords:** maize, siRNA, reproductive barrier, molecular evolution, silencing, gametophytic factors, pollen, silk, transmission ratio distortion, genome assembly

## Abstract

Three cross-incompatibility loci each control a distinct reproductive barrier in both domesticated maize (*Zea mays* ssp. *mays*) and its wild teosinte relatives. These 3 loci, *Teosinte crossing barrier1* (*Tcb1*), *Gametophytic factor1* (*Ga1*), and *Ga2*, each play a key role in preventing hybridization between incompatible populations and are proposed to maintain the barrier between domesticated and wild subspecies. Each locus encodes both a silk-active and a matching pollen-active pectin methylesterase (PMEs). To investigate the diversity and molecular evolution of these gametophytic factor loci, we identified existing and improved models of the responsible genes in a new genome assembly of maize line P8860 that contains active versions of all 3 loci. We then examined 52 assembled genomes from 17 species to classify haplotype diversity and identify sites under diversifying selection during the evolution of these genes. We show that *Ga2*, the oldest of these 3 loci, was duplicated to form *Ga1* at least 12 million years ago. *Tcb1*, the youngest locus, arose as a duplicate of *Ga1* before or around the time of diversification of the *Zea* genus. We find evidence of positive selection during evolution of the functional genes at an active site in the pollen-expressed PME and predicted surface sites in both the silk- and pollen-expressed PMEs. The most common allele at the *Ga1* locus is a conserved *ga1* allele (*ga1-Off*), which is specific haplotype containing 3 full-length PME gene copies, all of which are noncoding due to conserved stop codons and are between 610 thousand and 1.5 million years old. We show that the *ga1-Off* allele is associated with and likely generates 24-nt siRNAs in developing pollen-producing tissue, and these siRNAs map to functional *Ga1* alleles. In previously published crosses, the *ga1-Off* allele was associated with reduced function of the typically dominant functional alleles for the Ga1 and Tcb1 barriers. Taken together, this seems to be an example of an allele at a reproductive barrier locus being associated with an as yet undetermined mechanism capable of silencing the reproductive barrier.

## Introduction

Reproductive barriers restrict gene flow between populations, facilitating both neutral and adaptive divergence. When populations diverge, these barriers can lead to speciation (Kulmuni *et al*. 2020). Reproductive barriers can be classified into either prezygotic barriers, which function before fertilization, or postzygotic barriers, which function after fertilization. Prezygotic barriers are thought to generally be more complete, and thus more likely to lead to speciation, than postzygotic barriers (Baack *et al*. 2015; Christie *et al*. 2022). An important type of prezygotic reproductive barrier in plants is driven by postpollination/prefertilization interactions between the pollen, the male gametophyte, and the pistil, the female floral structure. Pollen–pistil interactions can reduce gene flow between populations in a variety of flowering plants, including maize (Broz and Bedinger 2021; Wang and Filatov 2023).

Indigenous peoples domesticated maize (*Zea mays* ssp. *mays*) over 9000 years ago in the Balsas valley region of Mexico (Piperno *et al*. 2009). At least 2 wild subspecies, the teosintes ssp. *Z. mays parviglumis* and *Z. mays mexicana*, played key roles in the origins of modern maize (Matsuoka *et al*. 2002; Yang *et al*. 2023). Farmers in Central America still cultivate maize alongside both of these taxa and other wild teosintes (Wilkes 1977). In some maize and wild teosinte populations, a group of 3 relatively common reproductive barriers controlled by the Gametophytic factor (GA) loci—*Tcb1*, *Ga1*, and *Ga2*—prevent gene flow between populations in only one direction to produce unilateral cross-incompatibility between populations (Kermicle 2006; Kermicle *et al*. 2006; Kermicle and Evans 2010).

**Fig. 1.**
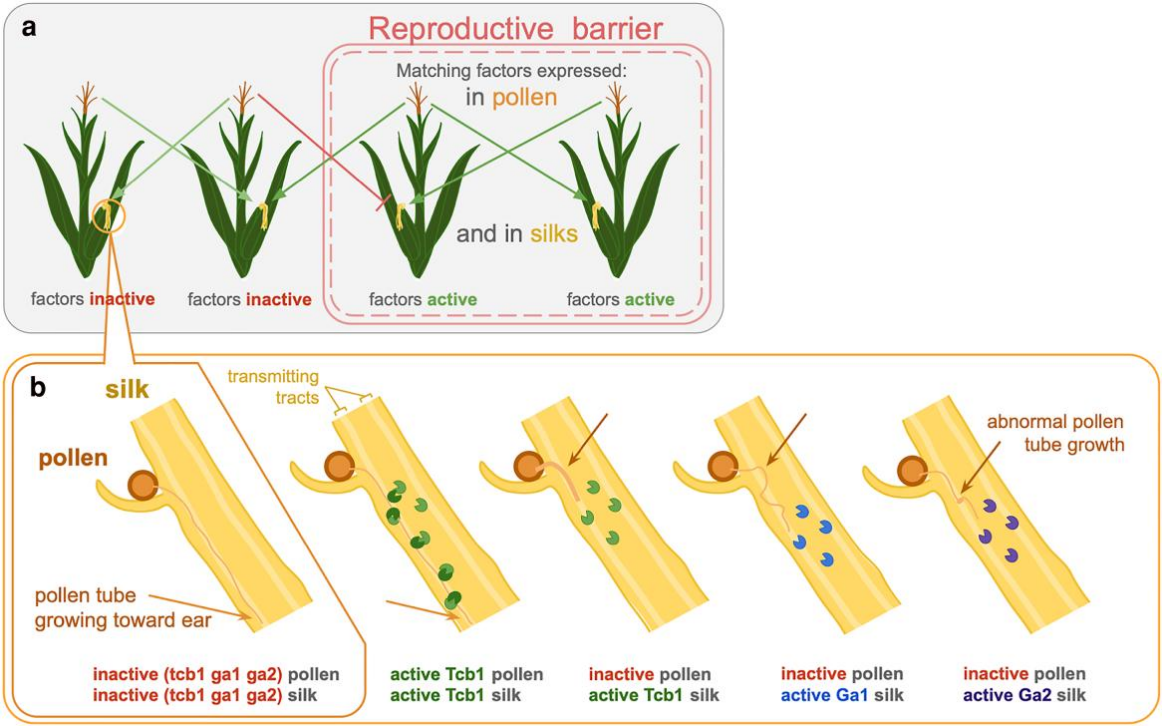
Incompatibility between GA inactive pollen and GA active silk can generate a reproductive barrier between *Zea mays* populations (a) when a gametophytic factor (GA) gene is active in the silk, only pollen with a matching active GA gene can grow normally down the silk toward the female gametophyte (light green arrow), which impedes the chances of fertilization by inactive GA pollen (dark red line) and generates a reproductive barrier. b) Diagram based in part on microscopy published by Lu *et al*. (2014): When silk and pollen both have no GA gene activity or both have matching active GAs, the pollen tube grows quickly down a transmitting tract in the silk toward the ear. Each of the 3 silk GAs impacts inactive GA pollen tube morphology differently. Tcb1 silk PMEs are shown in green, Ga1 in blue, and Ga2 in purple. Tcb1 pollen PMEs, shown in dark green, indirectly or directly interacts with silk PMEs.

The first of the 3 GA loci was characterized starting in 1901 when geneticists recorded phenotypic evidence, in the form of transmission distortion of the ratio of recessive sugary kernels, of a cross-incompatibility system in maize genetically controlled by a locus now known as *Gametophytic factor1* (*Ga1*) (Mangelsdorf and Jones 1926; Schwartz 1950; Correns 1901). The *Ga1* locus encodes 2 tightly linked gametophytic factors (genes) whose products interact after pollination but before fertilization. One gene generates an active prezygotic reproductive barrier in the female floral organ, the silk, and a matching second gene enables the male gametophyte, the pollen, to overcome that barrier (Fig. 1). The silk and pollen-expressed genes each encode distantly related pectin methylesterases (PMEs). PMEs play important roles in plant cell growth by enzymatically modifying cell wall pectin properties, impacting cell wall growth dynamics, especially in rapidly growing plant cells like those in both the pollen tube and silk tissues (Wallace and Williams 2017; Shin *et al*. 2021). When both the silk and pollen *Ga1* genes are active, the *Ga1* pollen tube can grow normally down the transmitting tract in the *Ga1* silk to eventually reach the female gametophyte, and fertilization can occur. In contrast, when the *Ga1* silk gene is active but the pollen gene is inactive, the *Ga1* silk impedes *ga1* pollen tube growth, possibly through the PME altering the integrity of the pollen tube cell wall and inhibiting directional growth. This inhibition reduces the chances of or prevents fertilization, producing the Ga1 reproductive barrier. The barrier only prevents gene flow from *ga1* to *Ga1* plants; in the opposite direction, GA active pollen can grow normally, although more slowly than GA inactive pollen, down a GA inactive silk (Lu *et al*. 2014). Study of *Ga1* is complicated by the fact that the locus contains a complex and polymorphic pattern of duplicated pseudogenes (Bapat *et al*. 2023).

**Fig. 2.**
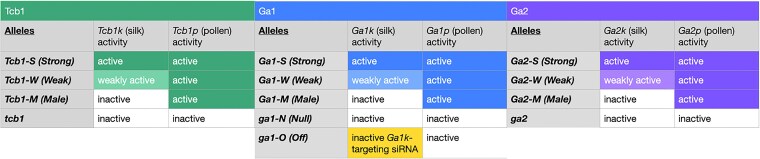
GA locus, allele, and gene nomenclature. Each GA barrier is controlled by a locus containing corresponding silk- and pollen-expressed genes. GA alleles can be categorized by activity of the PMEs, or factors, encoded by the genes in each locus. Here, we propose a new *ga1-O* allele, which is distinct from a fully inactive ga1 allele. Weakly active Tcb1, Ga1, and Ga2 barriers have been observed, but often under the control of alleles, which could be called strong in other genetic backgrounds. Alleles like these have sometimes been called *Ga1-W* and *Ga2-W*; to date, no *Tcb1-W* allele has been characterized.

Following the characterization of the Ga1 barrier and locus, maize geneticists identified and validated 2 additional GA loci, named *Teosinte crossing barrier1* (*Tcb1*) and *Ga2* (Burnham 1936; Brieger 1937; Evans and Kermicle 2001). Each locus functions similarly to *Ga1*, encoding a silk PME gene and a tightly linked, distantly related, matching pollen PME gene. For clarity, we call the genes active in the silk *Tcb1k*, *Ga1k*, and *Ga2k*, and the genes active in the pollen *Tcb1p*, *Ga1p*, and *Ga2p* (Fig. 2). In spite of the genetic and mechanistic similarity between loci, the 3 loci are distinct in the sense that each silk gene-encoded GA barrier can only be fully overcome by pollen with a matching active GA pollen gene, although pollen with a mismatched active GA gene is slightly preferred to pollen with no active GA genes (Lu *et al*. 2019). Additionally, the morphology of an inhibited wildtype pollen tube differs depending on whether the *Tcbk*, *Ga1k*, or *Ga2k* gene is active in the silk, suggesting that each silk gene may have a slightly different molecular function (Fig. 1) (Lu *et al*. 2014).

Although the GA loci have been studied for generations, the repetitive complexity of the loci and resulting recalcitrance to sequencing have long impeded research on the molecular evolution of the GA barriers. In the absence of high quality and complete gene sequence data, ecological and modeling research suggested different evolutionary histories for the GA loci. Because the barriers are commonly observed in sympatric teosinte populations, many authors have argued that the GA loci evolved to keep maize and teosinte distinct (Evans and Kermicle 2001; Zhang *et al*. 2023). However, population dynamics modeling work suggests that GA-like loci are largely unable to sustain a long-term crossing barrier between populations in an annual plant (Rushworth *et al.* 2022). Recent advances in sequencing have enabled the identification of all 6 types of genes controlling the gametophytic factors in maize, such that 1 reference gene sequence exists each for *Tcb1k*, *Tcb1p*, *Ga1k*, *Ga1p*, *Ga2k*, and *Ga2p* (Moran Lauter *et al*. 2017; Zhang *et al*. 2018, 2023; Lu *et al*. 2019; Chen *et al*. 2022). Simple sequence comparisons suggest the origins of the *Ga1k* and *Tcb1k* genes long predate the domestication of maize ∼9 k years ago (Bapat *et al*. 2023; Zhang *et al*. 2023) The evolution of GA loci is complicated further by evidence of the attenuation of incompatibility in some backgrounds (Demerec 1929; Nelson 1952; Ashman 1975; Lu *et al*. 2014; Lu *et al*. 2019; Goodman *et al*. 2021) and the observation of a complex series of highly repetitive haplotypes at the *Ga1* locus (Bapat *et al*. 2023).

Combined, these data motivate our detailed assessment of the diversity, function, and evolution of all the GA loci and how they may have impacted the evolution of *Zea*. Here, we work toward this goal by analyzing all 3 loci using long-read genome assemblies to improve reference gene models, identifying GA genes in more than 14 new species, and classifying the diversity of GA genes and loci. We establish a better understanding of the evolution and function of the GA system by estimating the timing of the evolution of the loci, testing for selection on genes and loci, and documenting the location of selected sites on predicted protein structure. Surprisingly, we find evidence for epigenetic silencing of the GA loci associated with an inactive *ga1* allele we call *ga1-Off*.

## Methods

### Genome assembly of maize line P8860

Maize line P8860, which creates and overcomes the Tcb1 and Ga2 barriers and overcomes the Ga1 barrier, was provided by Jerry Kermicle. High molecular weight DNA was extracted from young leaf tissue and sequenced via HiFi long-read sequencing on a Pacific Biosciences Sequel II at the UC Davis Genome Center. Reads were then assembled into a reference-quality genome using the Hifiasm assembler (Cheng *et al*. 2021), manually curated, and scaffolded using ALLMAPS (Tang *et al*. 2015). The P8860 genome assembly consists of 1,105 contigs with a mean contig length of 2,080,026 bp and a contig N50 of 91,160,284 bp for an estimated 99.90% coverage of the 2,300 Mbp genome. Genome features were annotated based on homology, resulting in complete versions of a total of 98.22% of the Poales obd10 BUSCO and 93.97% of the Liliopsida obd10 BUSCO genes (Manni, Berkeley, Seppey, Simão, *et al*. 2021; Manni, Berkeley, Seppey, Zdobnov, *et al*. 2021). CpG methylation was then inferred using the same HiFi reads and the software primrose (Hall *et al*. 2022), developed by Pacific Biosciences (accessed October 2022), which has since been replaced by an updated version of this tool called Jasmine (https://github.com/pacificbiosciences/jasmine/). Genome sequencing raw reads, assembly, and annotation are available at NCBI GenBank under project ID PRJEB86374.

### Gene identification

We used previously published and validated versions of the *Tcb1k*, *Ga1k*, *Ga1p*, and *Ga2p* genes as our reference version of each GA gene type, but the published *Ga2k* and *Tcb1p* gene references did not appear in the genome assemblies of maize lines we knew had full *Ga2* and *Tcb1* activity (McMullen *et al.* 2009; Moran Lauter *et al*. 2017; Zhang *et al*. 2018, 2023; Lu *et al*. 2019; Chen *et al*. 2022). To identify improved versions of the *Ga2k* and *Tcb1p* gene models, we searched within the *Ga2* and *Tcb1* regions of the P8860 genome for full-length gene models similar to the known GA silk and pollen genes, respectively. We identified new *Ga2k* and *Tcb1p* reference genes (see Results and Supplementary Fig. 1 for comparison between published gene models and our 2 reference gene models). These reference genes are present in all genome assemblies from plants known to create the Ga2 barrier and overcome the Tcb1 barrier, respectively. The new reference genes include signal peptides, have the conserved 1-intron structure for GA PMEs, and are expressed in plants and tissues known to create and overcome the barrier (Supplementary Table 3).

To identify GA genes across diverse Andropogoneae genomes, we BLASTed for GA reference gene sequences against the NAM v5 maize genomes, 4 Teosinte inbred lines (TILs), the 30 PanAnd project genomes, maize line Mo17, maize line W22, and our new genome assembly of maize line P8860 (Hufford *et al*. 2021; Woodhouse *et al*. 2021; Stitzer *et al*. 2025). We searched specifically for gene models supported by transcriptome annotations where available. We recorded genomic coordinates and pulled out CDS nucleotide and amino acid sequences for all high-quality hits (almost 100% match to the reference sequence used in our search query). In general, our BLAST hits for *Tcb1* and *Ga1* genes almost completely overlap due to similarity between the 2 loci, so we sorted *Tcb1* and *Ga1* hits by genomic location. We determined the genomic location of each locus by restricting just to the region between the area syntenic to the maize genes on either side of the Tcb1 and Ga1 loci on the maize consensus genetic map (available on MaizeGDB, accessed in 2023).

### Gene alignment and tree building

We aligned GA silk and pollen genes separately due to sequence dissimilarity between the 2 types of PMEs. To compare GA PMEs to Zea mays PMEs more broadly, we also included maize reference PMEs as an outgroup in all alignments and trees. We chose reference PMEs by searching the B73 genome annotation for genes with the Enzyme Code 3.1.1.11 for PME activity, and then selected a subset of those genes, which were annotated on MaizeGDB as being cited multiple times in published literature. We aligned these validated reference genes to each other, and we removed 2 PME genes that were too structurally different to be considered a useful reference. This resulted in 10 well documented reference PMEs that were used for comparative alignments (see Supplementary Fig. 1 for a list of these genes).

To focus the alignment on just the part of the gene that can be subjected to tests for positive or negative selection, we restricted the alignment to the CDS. Additionally, because signal peptides are under different selective pressures and evolve at a different rate than the amino acids that make up the mature protein, we used TargetP 2.0 to predict and cleave signal peptides at the beginning of all GA PMEs (Almagro Armenteros *et al*. 2019). We then aligned cleaved CDSs for all IDed gene models using Muscle5 with default parameter settings (Edgar 2022). Sequence alignments with all gene models, including those with stop codons in their CDS, were aligned without respect to codon position. Sequence alignments for only functional gene models, identified as those with identical or very similar sequence to gene models found in genomes from lines with GA silk or pollen function in the barrier phenotype, were also aligned without respect to codon position. Using cleaved CDS alignments, we assembled all gene trees in RAxML under a gammagtr model and bootstrapped all trees with at least 100 trials (Stamatakis 2014).

### Expression and methylation data

For all identified *Zea* GA gene models on main chromosomes (not scaffolds or contigs), we checked expression, methylation, and open chromatin (ATAC peak) status (Hufford *et al*. 2021; Woodhouse *et al*. 2021; Stitzer *et al*. 2025) (Supplementary Table 3). Expression is measured by mRNA transcript levels in RPKM, with RPKM > 5 constituting binary evidence of expression in contrast to no evidence of expression (Supplementary Table 3). Here, methylation is specifically 5-methylcytosine methylation across all 3 main plant contexts (CHH, CG, and CHG), while unmethylated regions are those with significantly lower CHG, or CHG and CG, methylation (see methods of Hufford *et al*. 2021 for details). We also checked the expression, inferred from RNAseq data, of the *Sorghum bicolor* Ga2 gene models, as this is the one other genome in our study with a wide range of publicly available RNAseq data aligned to a reference genome (Moreno *et al*. 2022). The ortholog of *Ga2k* (SORBI_3004G350500) is expressed in the inflorescence, seed, and drought-stressed root, while the ortholog of *Ga2p* (SORBI_3004g350400) is expressed in the inflorescence, anther, and pollen (Davidson *et al*. 2012; Wang *et al*. 2018; Varoquaux *et al*. 2019).

### GA loci age estimation

To estimate the age of the GA loci, we separately estimated the divergence times for functional silk and pollen copies of *Tcb1*, *Ga1*, and *Ga2*. Using MEGA, we ran a K2P model to calculate synonymous substitution rate between pairs of cleaved CDSs (Nei and Kumar 2000; Stecher *et al*. 2020; Tamura *et al*. 2021) (see Supplementary Table 2). We then used this substitution rate to calculate the divergence time by dividing by the 2 branches coming off of the shared ancestral node between the gene pairs and a constant average maize mutation rate (Clark *et al*. 2005), giving an estimate of generation time since divergence. Since maize is an annual species, we assumed that generation time was 1 year, and converted generation time to years. We compared pairwise generation times of functional genes from each locus, and averaged across unique pairwise comparisons to get an average divergence time between each type of gene that came from a duplication of a previous version of the locus (e.g. *Tcb1k* and *Ga1k* v *Ga2k* for the *Ga1* silk gene age estimate, and *Tcb1k* v *Ga1k* for the *Tcb1* silk gene estimate). For all pairwise comparisons, see Supplementary Table 2.

### Neutral allele frequency test

To test for evidence of selection at each locus, we compared the observed haplotype frequency spectrum to that expected under a simple neutral model. Each present observed haplotype (Fig. 4) was considered an allele in an observed allele frequency distribution (Supplementary Table 8). Expected allele frequency distributions were calculated for each locus using Ewen's sampling distribution, with *N* representing the total number of genomes with present observed haplotypes of the locus, and the population mutation rate *θ* chosen via a grid search (Supplementary Table 8). We used a multinomial test implemented in R with the package EMT (Menzel 2010) to calculate a *P*-value for the comparison of the observed haplotype distribution to that expected under the maximum likelihood value of *θ*.

## Results

### Refined models of gametophytic factor (GA) genes

**Fig. 3.**
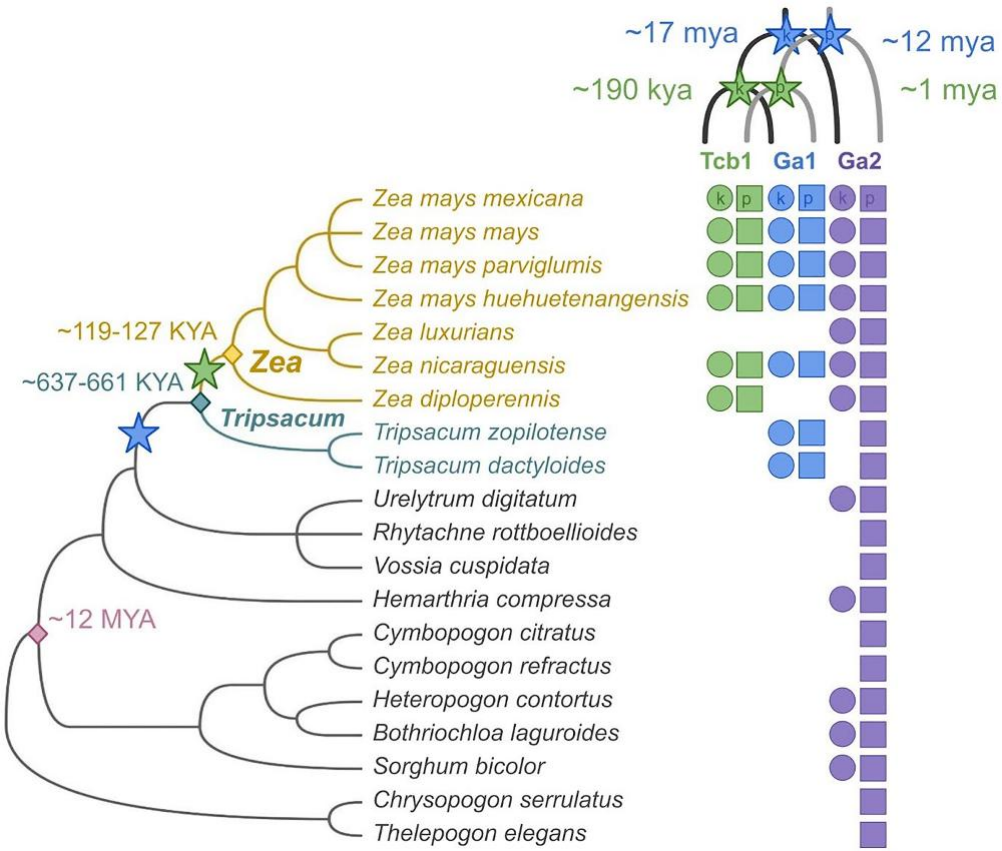
GA gene homologs are present in domesticated maize, 3 teosinte subspecies, and 16 other related grasses. Presence of GA genes is indicated by circles for silk-expressed (k) and squares for pollen-expressed (*p*) genes. Species divergence times are from (Welker *et al*. 2020; Chen *et al*. 2022), and the species tree is from (Grass Phylogeny Working Group III 2025). Divergence of *Ga1/Tcb1* from *Ga2* is indicated by a blue star and divergence of *Tcb1* from *Ga1* is indicated by a green star. Gene divergence times are based on sequence dissimilarity and Ks, see Supplementary Table 2. Trees visualized with iTOL (Letunic and Bork 2021).

Though individual reference gene sequences have been published for both silk and pollen PME genes for all 3 gametophytic factor loci, the full genomic regions for all 3 loci are largely unannotated across available genomes (Moran Lauter *et al*. 2017; Zhang *et al*. 2018; Lu *et al*. 2019; Chen *et al*. 2022; Zhang *et al*. 2023). To study the full sequences of all 3 GA loci, we identified their genomic regions in 52 genomes spanning *Zea* and related genera (Fig. 3; Supplementary Table 1). Our initial genome-wide BLAST search for the reference GA gene sequences did not find the published *Ga2k* and *Tcb1p* reference genes in genomes of plants known to produce the Ga2 barrier and overcome the Tcb1 barrier, functions encoded respectively by *Ga2k* and *Tcb1p*. We shifted to a synteny-based approach, using known genes from loci flanking each GA locus in the maize genomic map to identify the genomic coordinates of a large region that should contain the full GA locus. Using this approach, we were able to identify improved reference gene sequences for *Ga2k* and *Tcb1p.* These sequences share features of the other GA genes—including intron-exon structure, expression patterns, signal peptide presence, and amino acid similarity—and they are present and expressed in reproductive tissues of plants with barrier function (see methods). Our improved version of the *Ga2k* gene model is shorter than the previously verified *Ga2k* sequence sourced from a BAC, which includes 3′ sequence missing from genomes of maize plants that generate the Ga2 barrier (Supplementary Fig. 1) (Chen *et al*. 2022). Our *Tcb1p* gene model has a shifted intron position, which has little to no impact on amino acid sequence, but better allowed us to identify *Tcb1* loci because the nucleotide sequence is more consistent across species (Supplementary Fig. 1) (Zhang *et al*. 2023).

Our confidence in our improved gene models, and in our understanding of all 3 GA loci, comes in part from P8860, an inbred maize line with functional barrier loci (*Ga1-M*, *Tcb1-S*, and *Ga2-S*) introgressed from wild relatives (*Ga1-M* and *Tcb1-S* are from Collection 48703 of *Zea mays mexicana* (Kermicle and Allen 1990) and *Ga2-S* is from plant 3 in Collection 104 of *Zea mays parviglumis* (Kermicle and Evans 2010)). We assembled P8860 using long-read sequencing (see methods for assembly details). Our improved *Ga2k* and *Tcb1p* gene models, as well as previously published reference sequences for the other active genes, match sequences present in this genome (Supplementary Fig. 2). Many maize lines have no or only 1 active barrier and this is the first reference-quality genome assembly of a maize line with the Tcb1 barrier active.

### GA genes are present in diverse maize and wild relative genomes

To date, all 3 GA barrier phenotypes have only been reliably documented in subspecies of *Zea mays*. Using our updated gene models, we identified GA loci in 29 high-quality genome assemblies of maize lines and 23 genomes of related wild taxa from both *Zea* and 11 related *Andropogoneae* genera (Fig. 3). We focused interpretation on the presence of the locus in non-*Zea* taxa because absence of GA loci in these genomes reflect either true absence or false negatives arising from incomplete scaffolding. In general, these loci contain many truncated gene fragments and only a few full-length gene copies and the existing full-length gene models within the loci were unannotated in many of the genomes. All 3 loci, when present, are syntenic to the corresponding locus in *Zea mays*, and when silk genes are present, a tightly linked pollen gene is also found within the locus. These efforts represent the first comprehensive identification of *Ga1* and *Ga2* in non-*Zea* species.

Mapping the presence of the GA loci onto a phylogeny of the *Andropogoneae* tribe (Welker *et al*. 2020; Grass Phylogeny Working Group III 2025) indicates that *Tcb1* likely arose after the divergence of *Zea* from *Tripsacum* ∼650 K years ago, but before or around the time of the diversification of taxa within *Zea* around 170 K years ago (Fig. 3) (Chen *et al*. 2022). This clearly predates the divergence of the 3 wild subspecies of *Zea mays*, the teosinte ssp. *mexicana*, *parviglumis*, and *huehuetenangensis*, which first split 30–60 K years ago (Chen *et al*. 2022). Similar reasoning suggests that Ga1, present in *Zea* and *Tripsacum* but not in other genera, arose more than 650 K years ago, but likely after the Andropogoneae tribe arose ∼14 [9.89–17.97] million (M) years ago (Welker *et al*. 2020; Chen *et al*. 2022). We also estimated gene divergence times as an independent way of dating the origin of each locus. We used pairwise alignment of gene model sequences to calculate a synonymous substitution rate and estimated time since divergence, assuming 1 generation per year and a standard maize mutation rate of *μ* = 3.3*10^−8^ (Supplementary Table 2) (Clark *et al*. 2005). The gene divergence times are mostly consistent with the locus divergence timing estimates from the species phylogeny (Supplementary Fig. 3 and Table 2). We estimate that *Ga1k* diverged from *Ga2k* around 17 M years ago and *Ga1p* diverged from *Ga2p* around 12 M years ago, supporting the idea that *Ga1* arose from a duplication of *Ga2* ∼12–17 M years ago, consistent with previous work (Lu *et al*. 2019) (see Fig. 3). *Ga1* silk and pollen gene divergence times are similar, supporting the idea that silk and pollen genes were already tightly linked since before *Ga1* arose. In contrast, our estimates of the age of the *Tcb1* pollen and silk genes differ by an order of magnitude. While *Tcb1k* seems to have diverged from *Ga1k* ∼190 K years, consistent with silk gene presence in the species phylogeny, our estimate of divergence time between *Tcb1p* and *Ga1p* is ∼1 M years and predates the origin of the *Zea* genus. Although the timing of the *Ga2* duplication that led to *Ga1* is roughly concurrent with an ancient allopolyploidization event ∼10 M years ago in the Tripsacinae lineage (Wang *et al*. 2015), *Ga1* and *Ga2* do not share synteny beyond the local boundaries of the GA loci, suggesting that the Tripsacinae whole genome duplication was not the source of *Ga1* (Supplementary Fig. 4).

### GA loci exhibit high haplotypic diversity within Zea

**Fig. 4.**
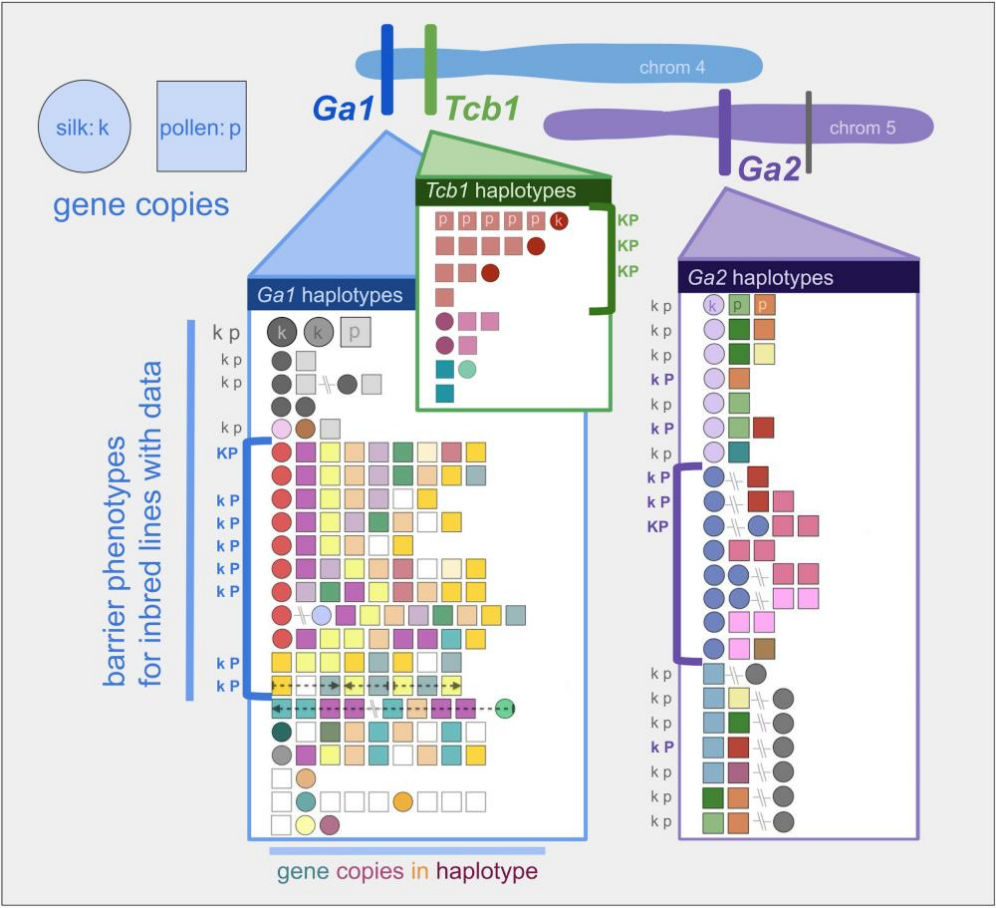
Haplotype diversity at each GA locus. Detailed view of haplotypes at the *Ga1* (blue) and *Tcb1* (green) loci on maize chromosome 4 and the *Ga2* (purple) locus on maize chromosome 5. Each row of shapes represents a sequence of full-length gene copies on a haplotype from 5′ to 3′, where circles are silk genes and squares are pollen genes. Shared gene copy color represents shared gene copy sequence. Gene copies with stop codons are gray, and gene copies found in only 1 haplotype are white. Hashed lines between gene copies represent more than a kilobase of distance between copies. Location of the distal nonfunctional silk gene copy found in some *Ga2* haplotypes is also marked on chromosome 5 in gray. Gene copies on a haplotype are all in the same direction, except where indicated by arrows. Known barrier activity is marked for inbred lines phenotyped for both silk (k) and pollen (*p*) activity, where bolded, colored, capital letters represent an active barrier (K) or ability to overcome the barrier (P). Haplotypes which seem similar to fully active haplotypes are grouped by brackets.

To assess the functional variation within and between each of the GA loci, we characterized the complete haplotypes of each GA locus in our sampled genomes by documenting the sequence similarity and gene order of full-length gene copies at each locus (Fig. 4). All 3 loci show variation in functional gene copy number, ranging from 0 to 2 for silk genes and 0 to 8 for pollen genes. While most haplotypes were found in a single individual, others were shared across up to 18 genomes. Given the observation that there are individual genotypes with functional gene sequences but nonfunctional barriers, we combined our sequence analysis of genome assemblies with functional genomic data for methylation (5-methylcytosine) and expression (RNAseq) available for many of these genomes to assess potential epigenetic differences. At all 3 loci, when the barrier is active the silk gene is unmethylated, and when the pollen can overcome the barrier the pollen genes are highly expressed, though sometimes display methylation in diploid tissue. Below, we discuss the specific observations for each locus individually.

Out of the 3 GA loci, *Ga2* exhibits the least haplotypic variation across genomes, and the locus is present in all genomes we studied. Across *Zea* genomes, many different haplotypes are present at low frequency, as expected under a neutral model of allele frequencies (Ewens sampling distribution with *θ* = 20; multinomial test *P*-value = 0.337831) (Supplementary Fig. 5 and Table 8). Unexpectedly, we discovered that a group of maize haplotypes missing a functional silk gene in the canonical *Ga2* locus is associated with an unmethylated, full-length copy of the *Ga2* silk gene nearly 50 Mb downstream of the *Ga2* locus (Supplementary Table 3). Each of these genomes shows a pollen gene at the syntenic position, but we find no evidence of structural rearrangements or genome duplications that can explain the distal silk gene location. Every *Ga2* silk gene copy with available methylation data, including distal *Ga2k* copies and *Ga2k* copies with premature stop codons, is unmethylated in diploid plant tissue (at CG, CHG, and CHH—“unmethylated” corresponds to UMRs identified in Hufford *et al*. 2021). *Ga2k* copies are expressed in a variety of tissues; including silk- and pollen-containing tissues and roots; with the exception of the *Ga2k* distal copies, which are not expressed in any tissue (Supplementary Fig. 6 and Table 3). Pollen genes exhibit variation in methylation in diploid tissues; a few *Ga2p* copies with stop codons are unmethylated, and about half of the full-length copies of *Ga2p* display TE-like methylation in leaf tissue, which is characteristic of some highly expressed maize pollen genes (Zeng *et al*. 2023, 2024). All *Ga2p* gene copies are expressed in pollen-containing tissue, and some *Ga2p* copies are also expressed in seeds (Supplementary Fig. 6 and Table 3).

*Ga1* haplotype diversity is dominated by 1 haplotype, which is found in most maize lines (depicted with larger shapes in the first row of Fig. 4, Supplementary Fig. 5). This conserved haplotype is composed of 2 silk gene copies and 1 pollen gene copy, all of which have premature stop codons. Many other haplotypes are present, but at low frequencies, and the overall frequency distribution deviates strongly from simple neutral expectations (*θ* = 38; multinomial *P*-value = 1 × 10^−7^) (Supplementary Fig. 5 and Table 8). Maize lines with documented Ga1 silk and pollen function (*Ga1-S* allele) or just pollen function (*Ga1-M* allele) all share a *Ga1k* gene copy but vary in *Ga1p* gene copy number and identity. Although silk expression data is not available for all *Ga1-S* lines, the silk gene in each of these lines is unmethylated and in a region of open chromatin. Pollen gene copies in these lines show TE-like methylation in leaf tissue, but are unmethylated and highly expressed in pollen (Zeng *et al*. 2024). Four maize lines with the *Ga1-M* allele (CML333, NC350, NC358, and Tzi8) show variable numbers of functional copies of the pollen gene and a highly methylated, unexpressed, full-length copy of the silk gene with promoter and coding sequences (CDSs) identical to expressed copies (Supplementary Fig. 5 and Tables 1 and 3). In another *Ga1-M* maize line that we examined (CML52), the full-length silk gene copy is unmethylated but lacks the ATAC signal typical of open chromatin that is found at active silk gene copies in other lines (Supplementary Tables 1 and 3). Observed expression of both silk and pollen functional *Ga1* genes are limited to reproductive tissues—anther and tassel tissues for pollen genes and silk for silk genes (Supplementary Fig. 6 and Table 3).

The *Tcb1* locus displays presence/absence variation across the *Zea* genus, and is absent in the vast majority of sequenced cultivated maize lines. The diversity of present *Tcb1* haplotypes reflects the species phylogeny, with sets of similar haplotypes shared within species. *Tcb1* allele frequencies match neutral expectation (*θ* = 8; multinomial *P*-value = 1) (Supplementary Fig. 5 and Table 8). Although published methylation data are unavailable for any of the published genomes containing *Tcb1*, we inferred CpG methylation across all 3 loci using HiFi reads from young leaf tissue that we also used for our P8860 genome assembly (Hall *et al.* 2022). Methylation at the *Ga2* and *Ga1* functional genes in P8860 are as expected—*Ga2k* is unmethylated while *Ga2p* and *Ga1p* both have CpG methylation—while at the active Tcb1 locus, the *Tcb1k* gene is unmethylated, and all 5 *Tcb1p* copies are methylated, which we expect based on the TE-like methylation we observed at active *Ga1p* genes. In lines with Tcb1 present, *Tcb1p* is expressed in the tassel, while *Tcb1k* shows expression in both root and reproductive tissue, similar to *Ga1k* (Supplementary Fig. 6 and Table 3).

### Evolution of the gametophytic factor genes and loci

To better understand the genetic relationship among gene copies and loci, we built separate phylogenies from full-length CDSs for silk and pollen gene models at all 3 loci (Supplementary Fig. 7). Silk and pollen tree topologies match each other, as expected for 2 genes which evolved with a shared function (Fryxell 1996). The species tree topology is also reflected in the gene trees, where within each locus, closely related genes are found in closely related species (Supplementary Fig. 7). The gene trees show that observed GA haplotypes consist of variable combinations of multiple distinct gene copies, identified by monophyletic subclades in the gene trees (Supplementary Fig. 7), and that many haplotypes with the same total gene copy number exhibit differences in the identity of the gene copies (Fig. 4).

An important exception is that, in both the silk and pollen gene trees, the GA genes found in the most common *Ga1* locus haplotype appear to be a conserved set of 3 full-length genes—2 noncoding silk genes and 1 noncoding pollen gene—each with CDSs containing distinct and conserved premature stop codons (Fig. 4, Supplementary Figs. 5 and 7). These nonfunctional genes are surprisingly diverged from validated functional *Ga1* genes and have an origin that is older than the split between functional *Ga1* and *Tcb1* genes. Specifically, based on sequence divergence, we estimate that these 3 older noncoding genes are roughly 610 and 750 K years old (silk) and 1.5 M years old (pollen), while the *Ga1* and *Tcb1* functional genes split ∼190 K years ago (silk) and ∼1 M years ago (pollen) (Supplementary Table 2) (Clark *et al*. 2005).

**Fig. 5.**
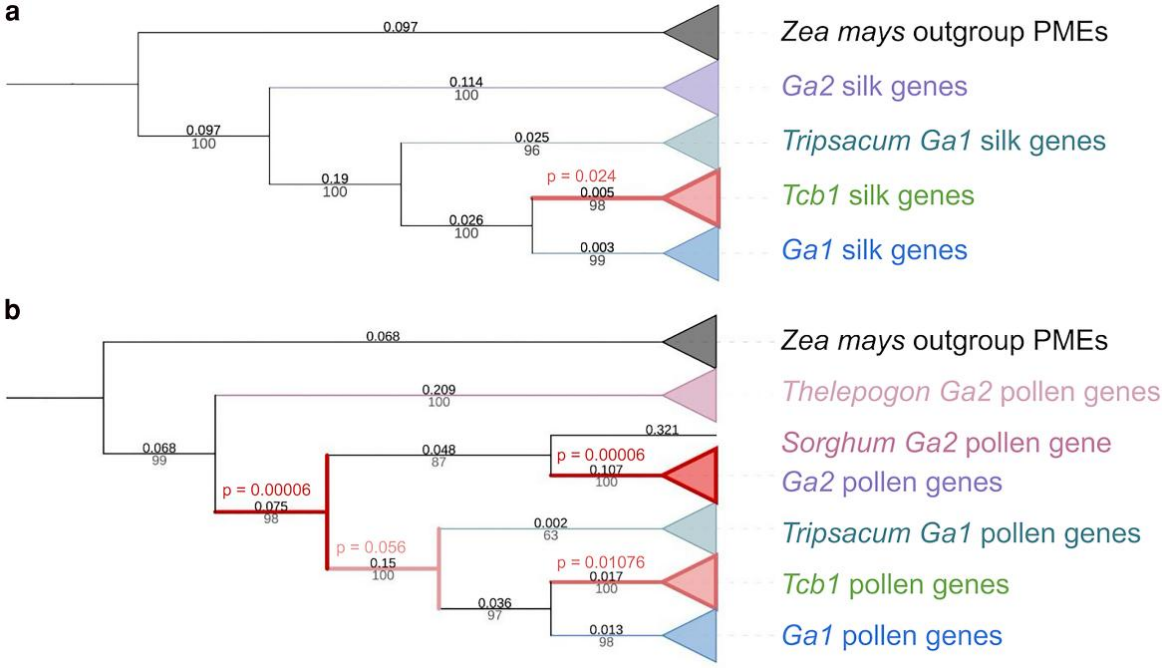
Signals of positive selection during the evolution of functional GA genes. GA genes display signals indicative of episodic positive selection (ω > 1) on gene tree branches subtending functional silk (a) and pollen (b) genes. Maximum likelihood trees were built in RAxML (Stamatakis 2014). Branches are labeled with lengths above in black and bootstrap values below in gray. Branches with significant evidence for positive selection are red, with additional *P*-value labels also in red on the (a) silk gene tree and (b) pollen gene tree. Trees visualized with iTOL (Letunic and Bork 2021).

We tested for episodic positive selection in both the silk and pollen gene trees by using a branch-site random effects model to check for elevated values of positive selection (*ω*) on all internal branches leading to divergence between GA gene types (Smith et al. 2015; Kosakovsky Pond *et al*. 2020) (Fig. 5). Both trees include an outgroup clade of 10 other functional maize PME genes, which represent all B73 maize PME genes that have the PME EC 3.1.1.11 and have been described in multiple papers. In the silk gene tree, the branch subtending the *Tcb1* silk genes shows significant change in selection (*P*-value = 0.024). In the pollen gene tree, the branch subtending all GA pollen genes and the branch subtending almost all *Ga2* pollen genes show significant change in selection (*P*-value = 0.00006 for both), as does the branch subtending all *Tcb1* pollen genes (*P*-value = 0.011). The pollen gene tree branch with the next most significant change in selection (*P*-value = 0.056) is the branch subtending all *Ga1* and *Tcb1* pollen genes.

### Molecular evolutionary analysis identifies patterns of constraint and adaptation on GA proteins

Because each GA locus generates a distinct barrier, we expect that positive selection resulted in specific amino acid changes which distinguish the barriers from each other. In particular, inactive pollen displays distinct morphology when growing down the silk depending on which silk-expressed gene—*Tcb1k*, *Ga1k*, or *Ga2k*—is active, so we expect the differences between GA silk amino acid sequences to drive the functional distinction between the barriers (Lu *et al*. 2014) (Fig. 1). To test for selection on individual amino acids that may control this impact of silk genotype on pollen growth, we used episodic positive selection tests on site changes from branches splitting the silk genes into distinct GA types (Murrell *et al*. 2012; Kosakovsky Pond *et al*. 2020). We identified 10 codons under significant positive selection on these branches of the silk gene tree (*P*-value < 0.1, the recommended *P*-value threshold for this test) (Supplementary Table 4). Because each GA silk protein interacts directly or indirectly with a paired GA pollen protein, we also checked whether the corresponding GA pollen genes may have evolved in concert. We found that 22 codons are under positive (*P*-value < 0.1) selection on the pollen gene tree (Supplementary Table 4).

**Fig. 6.**
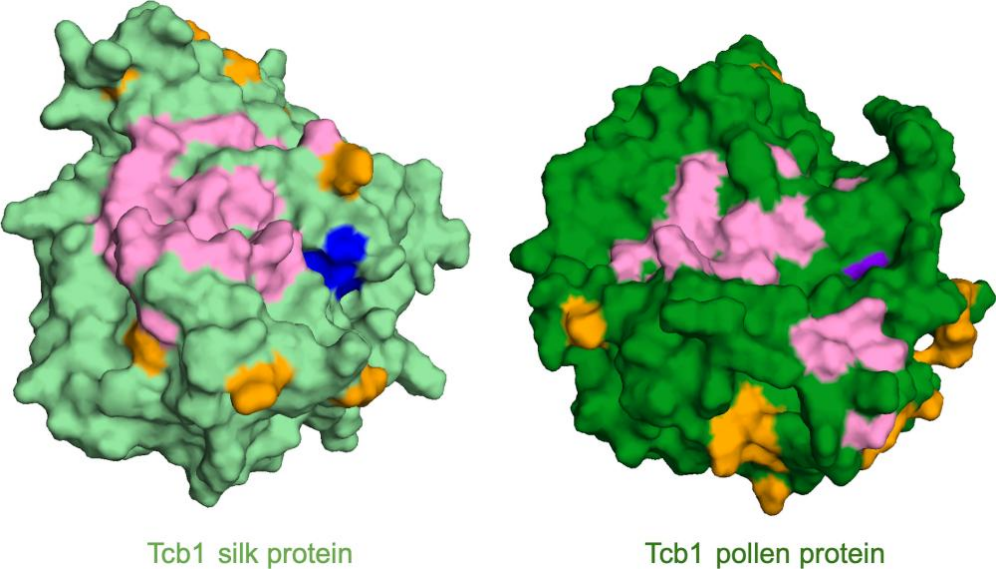
Sites likely under positive selection mapped onto 3D model of protein structure models of Tcb1 silk and pollen proteins colors represent inferred active sites without evidence of positive selection (blue), inferred active site with evidence of positive selection (purple), other sites with evidence of positive selection (orange), and location of predicted PME–PMEI interaction surface (pink). Sites displaying signals of positive selection are all on the surface and are not residues predicted to participate in PME inhibition via PME–PMEI binding. Active site residues (blue) were inferred via alignment to validated residues from (Johansson *et al*. 2002). PME–PMEI interaction residues are inferred based on alignments to validated residues from (Di Matteo *et al*. 2005). Structures predicted with AlphaFold2 (Jumper *et al*. 2021) and visualized in Pymol (Schrödinger n.d.).

Notably, 1 of the 4 active site residues predicted to catalyze the PME reaction corresponds to a codon under positive selection on the branch subtending all pollen GA PMEs, where all outgroup and silk GA PMEs have a Q and all pollen GA PMEs have an E (Fig. 6; Supplementary Table 4). This shift swaps the ancestral glutamine (Q) for a novel glutamic acid (E); this shift maintains the spatial volume but shifts the charge within the active site. While the activity shift caused is unclear, this is the only internal amino acid site under selection and is monophyletic for the change to GA pollen genes. Future work will need to ascertain its functional significance. Surprisingly, for both silk- and pollen-expressed genes, all of the sites under positive selection are on the surface of the predicted protein structures, where protein-protein interactions might occur. However, there is no overlap between the surface sites under positive selection and the sites where a known PME interaction with PMEI (Pectin methylesterase inhibitor) would occur (Di Matteo *et al*. 2005) (Fig. 5).

### Lines with a conserved, inactive *ga1* haplotype are associated with specific 24-nt siRNAs in developing pollen

**Fig. 7.**
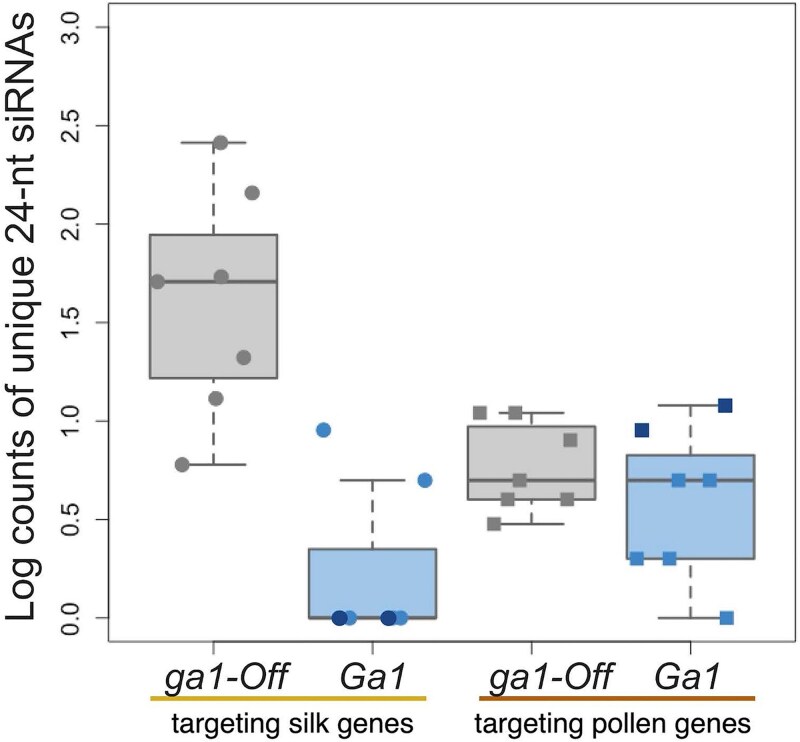
*Zea mays* premeiotic 0.4 mm anthers from maize inbred line plants homozygous for the *ga1-O* allele produce more unique 24-nt siRNA sequences targeting the GA silk gene sequences than inbred line plants homozygous for *Ga1-S* (dark blue) or *Ga1-M* (light blue) in anther RNAseq libraries. We observed no genotype-associated difference in the number of 24-nt siRNAs targeting GA pollen genes. Likewise, we observed no genotype-associated differences in nonanther tissues.

The conserved inactive *ga1* haplotype seems to serve some function; it is a haplotype that includes 3 highly conserved gene models that are in some cases expressed and unmethylated, and the haplotype is significantly more frequent than expected under a neutral model (see *Ga1* haplotype section). We propose that this haplotype is an *ga1* inactive allele with a nonbarrier function, which we name *ga1-Off* (*ga1-O*). To investigate a potential nonprotein-coding role of the *ga1-O* allele, we checked for an association between pollen siRNAs and the presence of this haplotype. Recent studies in maize have found pollen/anther-specific small RNAs may play an important role in pollen (Berube *et al*. 2024; Zhan *et al*. 2024). Using a database of siRNAs from the 0.4 mm (4 days prior to the start of meiosis in *Z. mays mays* when pollen is in an early mitotic stage of development) and 2 mm (late prophase I stage of meiosis in *Z. mays mays*) stages of anther development (Nakano *et al*. 2020), we searched for siRNAs in 3 maize inbred lines with *ga1-O* (B73, Oh43, and IL14H) and 3 genotypes with active versions of *Ga1* (*Ga1-S* maize inbred line HP301, *Ga1-M* maize inbred line NC358, and *Ga1-M mexicana* teosinte inbred line TIL25). We found that unique 24-nt siRNAs targeting reference GA silk gene sequences (*Tcb1k*, *Ga1k*, and *Ga2k*) are more abundant in 0.4 mm anthers of lines with the *ga1-O* allele compared to anthers with active *Ga1* alleles (*Ga1-S* and *Ga1-M*) (Welch's *t*-test, *P*-value = 0.03633) (Fig. 7). These siRNAs account for an average of 2 out of 20 million siRNA reads of all lengths in *ga1-O* lines and 118 in *Ga1* lines. In contrast, the number of siRNAs targeting reference pollen GA genes are similar across all genotypes (Welch's *t*-test, *P*-value = 0.5238), representing an average of 5 and 6 out of 20 million siRNA reads in *ga1-O* and *Ga1* lines. Out of the *Ga1* lines included, only NC358, which is *Ga1-M* with a methylated *Ga1k* gene, had any of these silk gene-mapping siRNAs. Sequence comparison using BLAST shows that many of the silk gene-mapping 24-nt siRNAs expressed in the *ga1-O* line B73 have SNPs unique to the *ga1-O* allele that are not found elsewhere in the B73 genome, indicating that the *ga1-O* allele is likely the source of 24-nt siRNAs and *Ga1* alleles the target (Supplementary Figs. 8 and 9) (Nakano *et al*. 2020). The 24-nt siRNAs we found to be enriched in the *ga1-O* lines are unphased (Supplementary Fig. 8). In anther tissues, 24-nt siRNAs were the only length of siRNAs that showed a difference in number across genotypes, and the difference was only significant in the 0.4 mm and not the 2 mm (Welch's *t*-test, *P*-value = 0.088) anther stage (Supplementary Table 5). In published siRNA data from maize inbred lines, 24-nt siRNAs mapping to the full-length silk gene copies in the *ga1-Off* region of the genome are present in 0.4, 0.7, 1.0, 1.25, 1.5, 2.0, 3.0, 4.0, and 5.0 mm long anthers, spanning thirty days of pollen development from the early mitotic to the binucleate microspore stage of pollen development (Supplementary Fig. 10) (Zhai *et al*. 2015; Nakano *et al*. 2020). Phased 24-nt siRNAs generated by the somatic tapetal cells are transported into meiotic pollen cells (Zhou *et al*. 2022). Similarly, it may be possible that the unphased 24-nt siRNAs present in 0.4 mm anthers, during a stage of development before the tapetum has formed, may be generated by the somatic diploid anther tissues and either persist or continue being generated for weeks of anther development until at least the 5 mm stage, eventually impacting the developing haploid pollen (Chow and Mosher 2023; Zhan *et al*. 2024). We did not observe a difference in the number of siRNAs mapping to GA silk or pollen genes across genotypes in leaf or internode tissues (Nakano *et al*. 2020).

## Discussion

### Gametophytic factors may have contributed to *Zea* diversification

The gametophytic factor loci *Ga2*, *Ga1*, and *Tcb1* generate reproductive barriers in *Zea mays* and have been proposed to be subspecies barriers (Evans and Kermicle 2001; Chen *et al*. 2022). Recent modeling work, however, suggests that the individual impact of any one of these loci is unlikely to prevent gene flow between species for more than 10,000 generations (Rushworth *et al.* 2022). This is partially due to the inability of GA-like loci to maintain reproductive isolation between distinct populations that come into contact, likely precluding their role in maintaining species boundaries (Rushworth *et al.* 2022). The presence of putatively functional copies of each barrier gene in more diverged genera, along with our sequence-based estimates of divergence times of individual genes, provides clear evidence that even the youngest of these loci, *Tcb1*, did not evolve recently to prevent gene flow from domesticated maize into teosinte.

One possible role for the Tcb1 barrier could have been to maintain distinction between diverging populations during the *Zea* genus diversification. Both the date of the *Tcb1k* origin around the time of *Zea* genus diversification, as also observed by Bapat *et al*. (2023) and Chen *et al*. (2022) and the pattern of Tcb1 diversity across species are consistent with this role. *Tcb1k* is also completely absent in maize lines that do not generate a barrier; therefore, selection on this gene likely reflects selection on the functional barrier. Additionally, we observe positive selection on the branch leading to *Tcb1k* genes, suggesting that the role of Tcb1 as a species barrier was under selection before and as *Zea* was diversifying (see Fig. 5). Our observation of a full-length putatively functional copy of the *Tcb1k* gene in *Z. nicaraguensis* is evidence that the Tcb1 barrier may currently play a role outside of the *Z. mays* subspecies. Conflicting with a possible role for Tcb1 barrier in species diversification via reproductive isolation, the age of *Tcb1p* is relatively ancient; we estimate *Tcb1p* diverged from *Ga1p* around 1 M years ago, significantly predating the diversification of the genus ∼170 K years ago (Chen *et al*. 2022). Our relatively old age estimate for Tcb1p is based on synonymous substitution rate, which may be artificially elevated due to recent positive selection on nonsynonymous and linked synonymous sites; for example, multiple sweeps rather than age could explain the high divergence of Tcb1p from Ga1p (Fig. 5). Further research is needed to functionally validate these and other putatively functional GA gene copies found in non-*Z. mays* genomes. However, the fact that functional silk-expressed gene copies are shared between *Z. mays* and other *Zea* species at all 3 barrier loci strongly supports the idea that these barriers are functional across the genus.

### Potential origins of the gametophytic factor reproductive barriers

The anatomy of maize and related grasses may have facilitated the retention of pollen–pistil barrier loci. Compared to related taxa, *Tripsacum* and especially *Zea* species have unusually long silks (stigmas) (Supplementary Fig. 11 and Table 6). After pollen germinates on a silk, the pollen tube grows into and down the remaining length of a transmitting tract inside the silk tissue to reach the female gamete. In species with longer transmitting tracts, this could present more opportunity for the pollen tube growth pattern and rate to become an important driver of fitness. Consistent with this idea, after the origin of the Ga2 barrier, duplicates like those which led to Ga1 and Tcb1 seem to only have been retained in the sister genera *Zea* and *Tripsacum* and not in related species with shorter silks. Long silks may have enabled GA barriers to evolve regardless of whether or not GA barriers conferred an adaptive benefit; the barriers are transiently reinforcing (Rushworth *et al.* 2022), so they may have been able to persist on short timescales just by selfishly excluding pollen from outside populations. Long silks may have also increased the impact of pollen-silk interactions, including interactions between PMEs and other proteins, on fitness. One possible interaction protein could be a linked and silk-expressed pathogenesis-related protein called *ZmPRP3*, which has been proposed as a third component of the *Ga1* locus that enables pollen tube growth (Wang *et al*. 2022). This would suggest a potential overlap with the role of silk PMEs in mediating pathogen response, and introduces the possibility that the GA PMEs may have originally played a role in impeding pathogen growth down the silk (Begcy *et al*. 2024). However, to date no research has shown a role of GA loci in silk pathogen resistance, and *ga1* silks were not more susceptible to silk-invading fungal pathogens than *Ga1* silks (Begcy *et al*. 2024).

The GA barriers are often compared to pistil–pollen self-incompatibility mechanisms, and it has been suggested that the GA loci have origins in an ancestral self-incompatibility function (Kermicle and Evans 2005; Dresselhaus *et al*. 2011). Our results do not clearly support this hypothesis. Although many haplotypes have GA pollen genes and no corresponding GA silk gene, this is not evidence that the pollen function evolved first; we expect there to be strong selection against the opposite configuration of only a functional silk gene and no functional pollen gene, which would lead to incompatibility with all other plants. Better evidence for the ancestral function of these genes comes instead from expression patterns of the current functional gene copies. In both maize and *Sorghum bicolor*, we see expression of the *Ga2* silk-expressed gene in both the silk/stigma and in the root (Supplementary Table 3) (see methods). PMEs play an important role in cell wall formation, growth, and maintenance, and diverse PMEs are present in organisms as distantly related as bacteria and plants (Markovič and Janeček 2004; Shin *et al*. 2021). Although various PMEs work in concert to coordinate cell wall integrity in growing plant tissues, the age of the PME family means that these proteins are in many cases distantly related to each other despite their shared protein function. The silk and pollen PMEs encoded by the gametophytic factors are not closely related. In general, the exact mechanism of the interaction between GA PMEs is unclear, but there is no evidence supporting the idea that self-incompatibility was a role for these PMEs as they evolved.

Further complicating interpretation of the evolution of the GA silk and pollen PME interaction is the fact that interaction surfaces of the GA PMEs seem to have been under selection (Fig. 5). Although direct interaction of 2 PMEs has never been documented, PMEs often bind to PME inhibitors (PMEIs), which typically include a functional PME domain and an inhibitor domain (Di Matteo *et al*. 2005). To date, other direct interactions between PMEs and other types of plant proteins have not been documented. The prevalence of PMEs and PMEIs in the silk and pollen tube provides the opportunity for many PME–PMEI interactions, including in complexes of more than 1 PME and PMEI. For example, previous research has implicated an additional maize PME, *ZmPME10.1*, as a component of a complex in which the *Ga1* and *Ga2* silk and pollen PMEs interact (Zhang *et al*. 2018; Chen *et al*. 2022). Additionally, interactions of the GA PMEs with other proteins may be important. This is supported by the fact that all sites we found to be under positive selection are surface sites, but none overlap with the predicted site of PME–PMEI interaction.

### The *ga1-O* allele may function to suppress active gametophytic factors

We were surprised to find that the most common haplotype of the *Ga1* region, which we call the *ga1-Off* allele, is an inactive *ga1* haplotype present in lines which up until now have been considered to be fully inactive because they have no Ga1 barrier function (Fig. 4). Within this haplotype, the putative CDSs of the 3 full-length gene models, including stop codons, are highly conserved despite the fact that all 3 models seem to be noncoding. All 3 genes diverged from *Ga1* between 0.6 and 1.5 M years ago, well before the *Zea* genus diversification, and before the divergence between *Ga1* and *Tcb1*. The high frequency of the *ga1-O* haplotype in the population strongly suggests that this is not the result of a recent expansion of a neutral allele (*P*-value = 1 × 10^−7^, see Results). Instead, we suggest the genes are conserved because this haplotype likely functions as an allele which can suppress active silk gametophytic factors.

The identification of a *ga1-Off* allele helps to explain prior observations that an active *Ga1* barrier allele can be suppressed when crossed into certain backgrounds. In 2 previous studies, the action of a popcorn-derived *Ga1-S* allele conferred differing barrier strength after backcrossing into different inactive *ga1* backgrounds (Nelson 1952; Ashman 1975). *Ga1-S* shows dominance when introduced into maize dent inbred Hy, but the barrier strength is significantly reduced when introduced to 2 different popcorn inbreds, Sg1533 and Sg18 (Nelson 1952; Ashman 1975). Using previously published SNP data from the Ames 282 panel, a set of diverse maize lines including many used in important maize literature and pedigrees throughout the decades, we found that Sg1533 and Sg18 likely both carry the B73-like *ga1-O* allele, while Hy carries a B97-like *ga1* allele (Flint-Garcia *et al*. 2005; Bukowski *et al*. 2018) (Supplementary Fig. 12). The B97-like *ga1* allele, in contrast to the *ga1-O* allele, is not notably common or conserved and seems to behave as a truly nonfunctional ga1 allele, so we call this *ga1-Null* or *ga1-N*. We argue that the reduced activity of the *Ga1-S* allele in these experiments can be ascribed to the *ga1-O* v *ga1-N* identity of the inactive *ga1* allele in a heterozygous background.

The *ga1-O* gene copies are equally related to functional *Ga1* and *Tcb1* genes (Supplementary Fig. 3 and Table 2). Consistent with this phylogenetic relationship, *ga1-O* seems to also silence active *Tcb1*. After 10 generations of backcrossing a *Tcb1-S* allele into W22, an inbred that carries the *ga1-O* allele, the teosinte Tcb1 barrier activity was fully suppressed in 2 independent lineages (Lu *et al*. 2014). When these lines with suppressed Tcb1 barriers were crossed into backgrounds lacking the functional *Mop1* RNA-dependent RNA polymerase, some of the offspring regained Tcb1 barrier function and *Tcb1k* expression (Lu *et al*. 2019). Additionally, in sympatric populations of maize and teosinte, the Tcb1 barrier has only been observed in populations where *Ga1* is at least partially active (*Ga1-S* or *Ga1-M*) (Kermicle 2006; Kermicle *et al*. 2006) (Supplementary Table 7). Because *ga1-O* is by far the most common *ga1* allele and Tcb1 is active in most of these teosinte populations, the absence of Tcb1 barriers here may indicate that *ga1-O* is suppressing Tcb1 activity. It is possible that *ga1-O* may similarly regulate Ga2, based on sequence similarity to Tcb1 and Ga1, and 24-nt siRNA sequence match to *Ga2k* (Supplementary Table 5), and anecdotal evidence of Ga2 barrier suppression. However, the *Ga1* locus is 10–100 times more diverged from the *Ga2* compared to the *Tcb1* locus (see Results), Ga2 and Ga1 barriers are active in populations with *ga1* alleles (Kermicle *et al*. 2006; Kermicle and Evans 2010) (Supplementary Table 7), and we found no evidence of *Ga2k* genes being methylated in any background, so the Ga2 locus may be under a different type of regulation. Future experiments will be required to establish a strong causal connection between the *ga1-O* locus and silk-expressed barrier function at any of the 3 gametophytic factor loci.

The exact functional and mechanistic difference between the 2 types of inactive *ga1* alleles we observed, *ga1-O* and *ga1-N*, is unclear from our experiments. Previously, Ashman suggested a similarity between what he termed the “suppression” of the *Ga1-S* allele and outcomes expected if either a dominant modifier or a paramutation system was at play (Ashman 1975). Without experimental data testing the behavior of the Ga1 barrier in plants heterozygous for *Ga1/ga1* alleles but with an otherwise controlled genetic background, silencing directed by linked modifying alleles cannot be ruled out. However, we do observe parallels between our findings and more current understandings of silencing mechanisms, including paramutation, which could control the suppression. If silencing is involved, the silencing mechanism may be controlled by 1 or more linked modifying loci or be directly controlled by the *ga1-Off* allele itself. The methylated status of *Ga1k* genes in *Ga1-M* maize lines where variable methylation in gene bodies is correlated with expression (Hufford *et al*. 2021), the fact that *ga1-Off* is associated with 24-nt siRNAs unique to the *ga1-Off* sequence, and the potential involvement of 24-nt siRNAs in siRNA-mediated RdDM pathway that could methylate GA silk genes all support the idea that *ga1-Off* is involved in some silencing mechanism. In theory, the silencing of *Ga1k* could explain the apparent transformation of a *Ga1-S* allele into a *Ga1-M* allele despite the presence of genetically identical *Ga1k* genes in lines with active and inactive Ga1 barriers.

Importantly, we have not established a strong causal link between the *ga1-O* locus and methylation of silk *Ga1* genes, nor have we established a strong causal link between methylation of silk Ga1 genes and activity of the barrier. To determine whether Ga1 is truly silenced by a linked allele, a paramutation system, or a different silencing mechanism would require rigorous testing of the behavior of *Ga1-S* and *ga1-O* alleles in controlled backgrounds and across generations.

## Conclusion

For all 3 maize gametophytic factors, we documented haplotype and gene diversity, identified sites under positive selection, and estimated the timing of gene and locus divergence. We also sequenced a maize line with all 3 barriers at least partially active, which allowed us to observe a correlation between gene methylation and barrier activity at all 3 silk genes. This silk gene methylation may be regulated by pollen-expressed 24-nt siRNAs created by the *ga1-O* allele. Future work would be needed to functionally validate the role of the *ga1-O* allele by establishing a causal relationship between the allele, the associated 24-nt siRNAs, and the silencing of the Tcb1, Ga1, and Ga2 barriers.

## Supplementary Material

iyaf085_Supplementary_Data

## Data Availability

The Supplementary Figures file has all Supplementary Figures, including alignments of old and new reference GA genes, gene trees, 24-nt siRNA phasing scores across *ga1-Off* gene copies, species tree with silk length data, and SNP-based trees for the *Ga1* locus across diverse maize lines. Supplementary Table 1 has the CDS and genomic coordinates for all full-length GA loci gene copies we identified. Supplementary Table 2 includes genetic distance and corresponding gene age estimates. Supplementary Table 3 has expression data summary, methylation status, ATAC peak presence or absence, and genomic coordinates for identified gene copies in *Zea* genomes. Supplementary Table 4 has results from HyPhy MEME selection testing, detailing sites under positive selection. Supplementary Table 5 has 24-nt siRNA counts from 0.4 to 2 mm maize and teosinte anthers. Supplementary Table 6 has data on silk length measurements and sources. Supplementary Table 7 is a summary of published GA activity in a set of sympatric maize and teosinte populations. Supplementary Table 8 is observed and expected GA allele frequencies under a Ewens sampling. The P8860 genomic sequence is publicly available at NCBI GenBank under project ID PRJEB86374, and the genomic sequence and annotation are hosted publicly at MaizeGDB and directly accessible for download at https://download.maizegdb.org/Zm-P8860-REFERENCE-TeoGa-1.0/. Supplemental material available at GENETICS online.
