# Supplementary material for: Molecular evolution of a reproductive barrier in maize and related species
Source: Genetics. 2025 May 8;230(3):iyaf085. doi: 10.1093/genetics/iyaf085 (PMC12239200; doi:10.1093/genetics/iyaf085)
Supplement: iyaf085_Supplementary_Data [file iyaf085_supplementary_data.zip › Supplemental_Figures_GENETICS-2025-308042.pdf]

Supplemental Figures

Molecular evolution of a reproductive barrier in maize and related species

Elli Cryan, Garnet Phinney, Arun S. Seetharam, Matthew M.S. Evans, Elizabeth A. Kellogg, Junpeng Zhan, Blake C. Meyers, Daniel J. Kliebenstein, Jeffrey Ross-Ibarra

Supplemental figure 1: Alignments of published and new GA reference genes

Supplemental figure 2: Gene trees of putatively functional GA genes

Supplemental figure 3: Simplified gene trees with estimated divergence times

Supplemental figure 4: *Ga2* and *Ga1* are not duplicates from the Tripsacinae WGD

Supplemental figure 5: Detailed haplotype diversity at each GA locus with haplotype frequencies and line and species names labeled

Supplemental figure 6: Expression anatograms by gene

Supplemental figure 7: Gene trees of all full-length GA genes

Supplemental figure 8: 24nt siRNA hits and phase scoring for each *ga1-O* ‘gene’ in B73

Supplemental figure 9: 0.4mm anther 24-nt siRNAs from ga1-Off line B73 map uniquely to the ga1-Off Ga1k gene copies of the B73 genome but also to the functional Ga1k gene sequence from Ga1-S line HP301

Supplemental figure 10: *ga1-O*-matching 24nt siRNA expression across stages of maize anther development

Supplemental figure 11: Stigma length and GA locus PAV on Andropogoneae species tree

Supplemental figure 12: SNP trees for GA loci across diverse maize lines

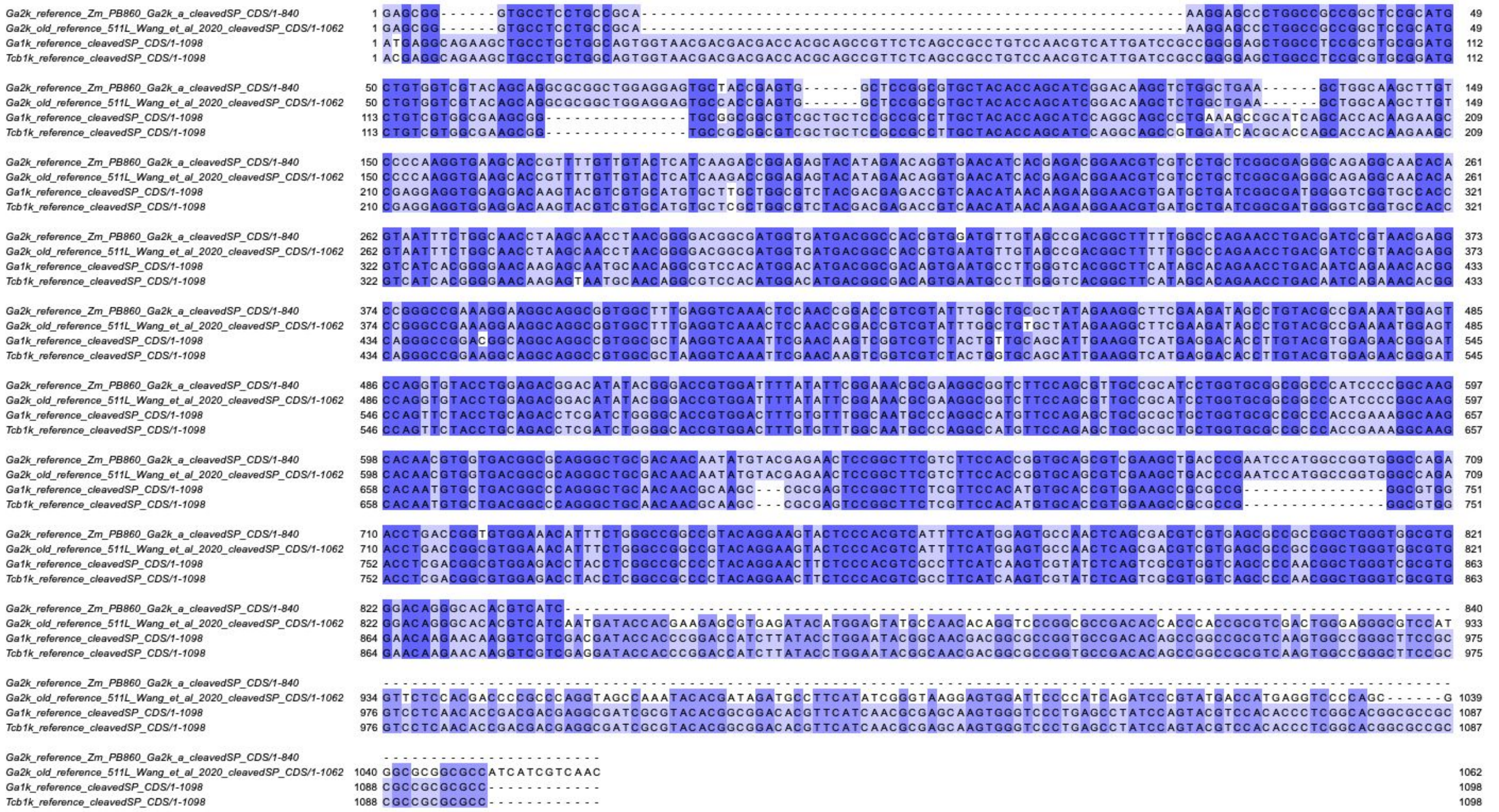

Supplemental Figure 1a
Alignment of published and new GA silk reference gene enzyme-coding sequences
Alignment of GA silk reference gene sequences without N-terminal signal peptide-encoding sequences. Our new *Ga2k* sequence has a shorter CDS. Codon-aware nucleotide alignments of nucleotide CDSs were made in muscle and visualized in Jalview. Color shows percent shared identity across sequences.

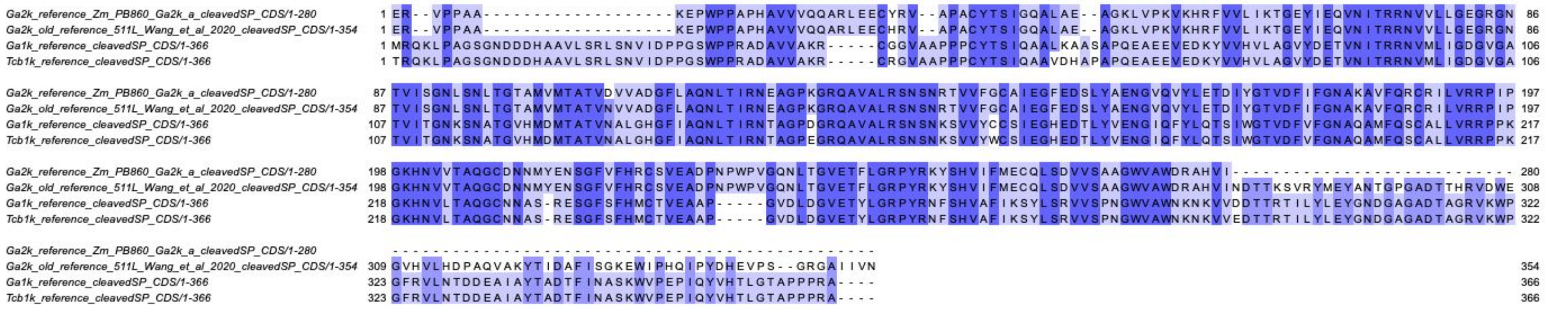

Supplemental Figure 1b
Alignment of published and new GA silk reference gene enzyme sequences
Alignment of GA silk reference gene amino acid sequences without N-terminal signal peptide sequences. Our new *Ga2k* sequence has a shorter CDS. Alignments were made in muscle and visualized in Jalview. Color shows percent shared identity across sequences.

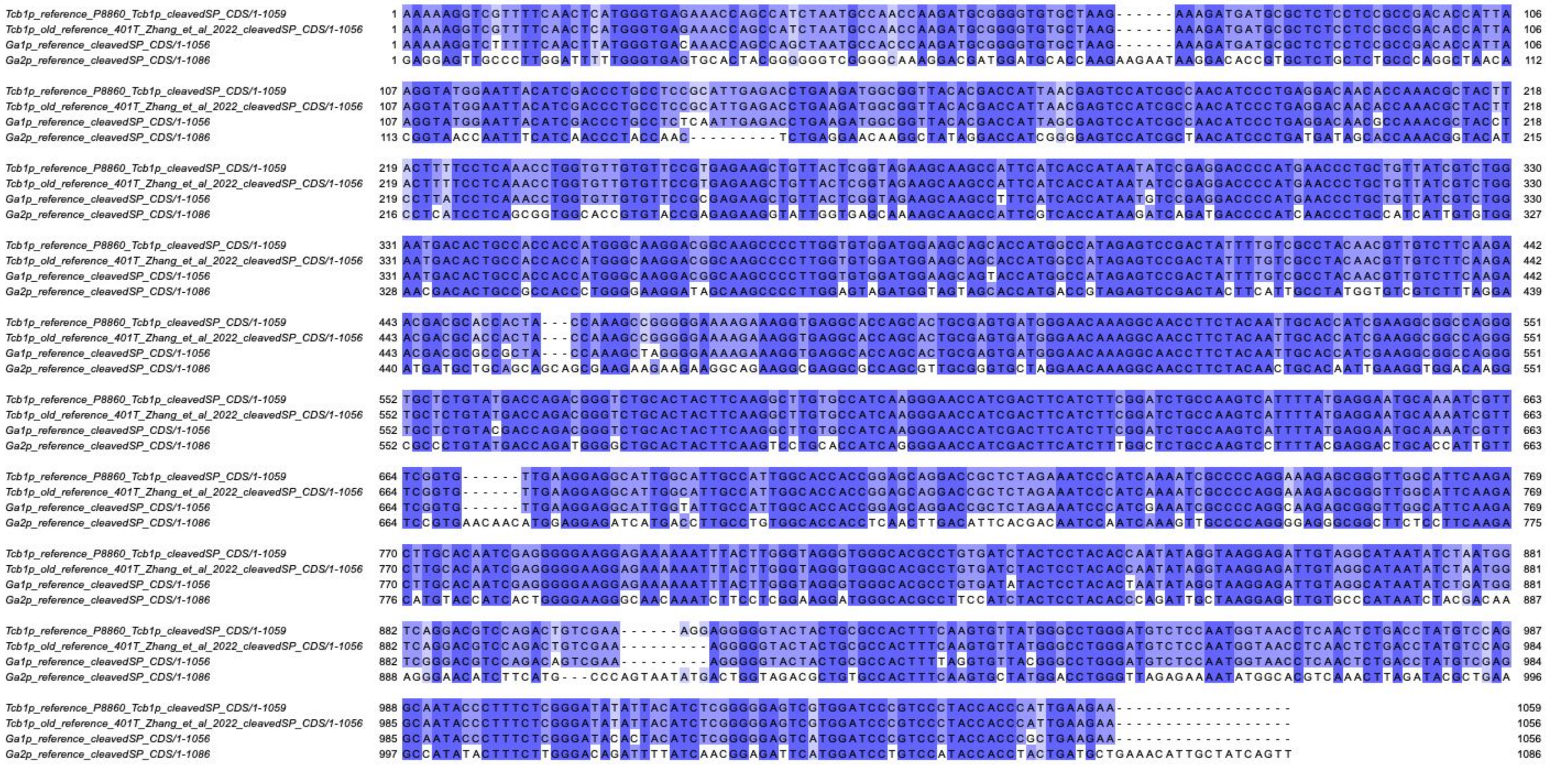

Supplemental Figure 1c
Alignment of published and new GA pollen reference gene enzyme-coding sequences

Alignment of GA silk reference gene sequences without N-terminal signal peptide-encoding sequences. Our new *Tcb1k* sequence has a shorter intron, which leads to a single codon change at nucleotide 904-906. Codon-aware nucleotide alignments of nucleotide CDSs were made in muscle and visualized in Jalview. Color shows percent shared identity across sequences.

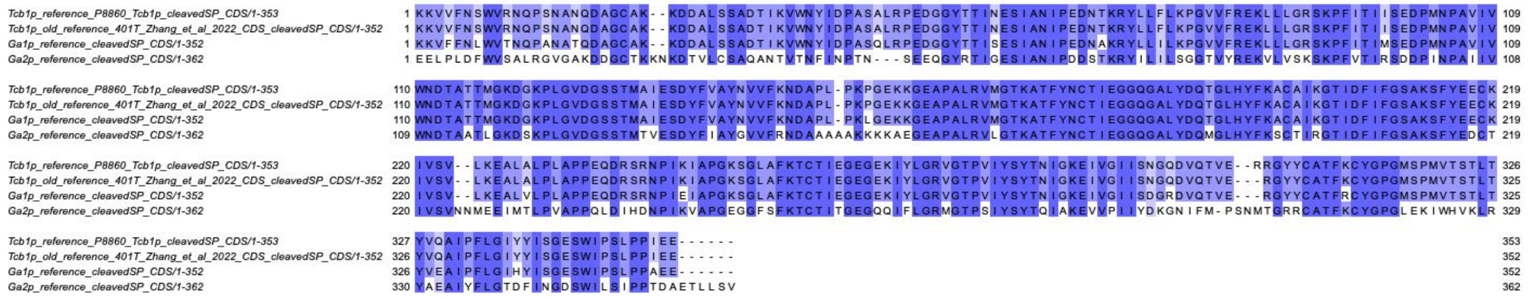

Supplemental Figure 1d
Alignment of published and new GA pollen reference gene enzyme sequences

Alignment of GA pollen reference gene amino acid sequences without N-terminal signal peptide sequences. Our new *Ga2k* sequence has an additional arginine at amino acid 302. Alignments were made in muscle and visualized in Jalview. Color shows percent shared identity across sequences.

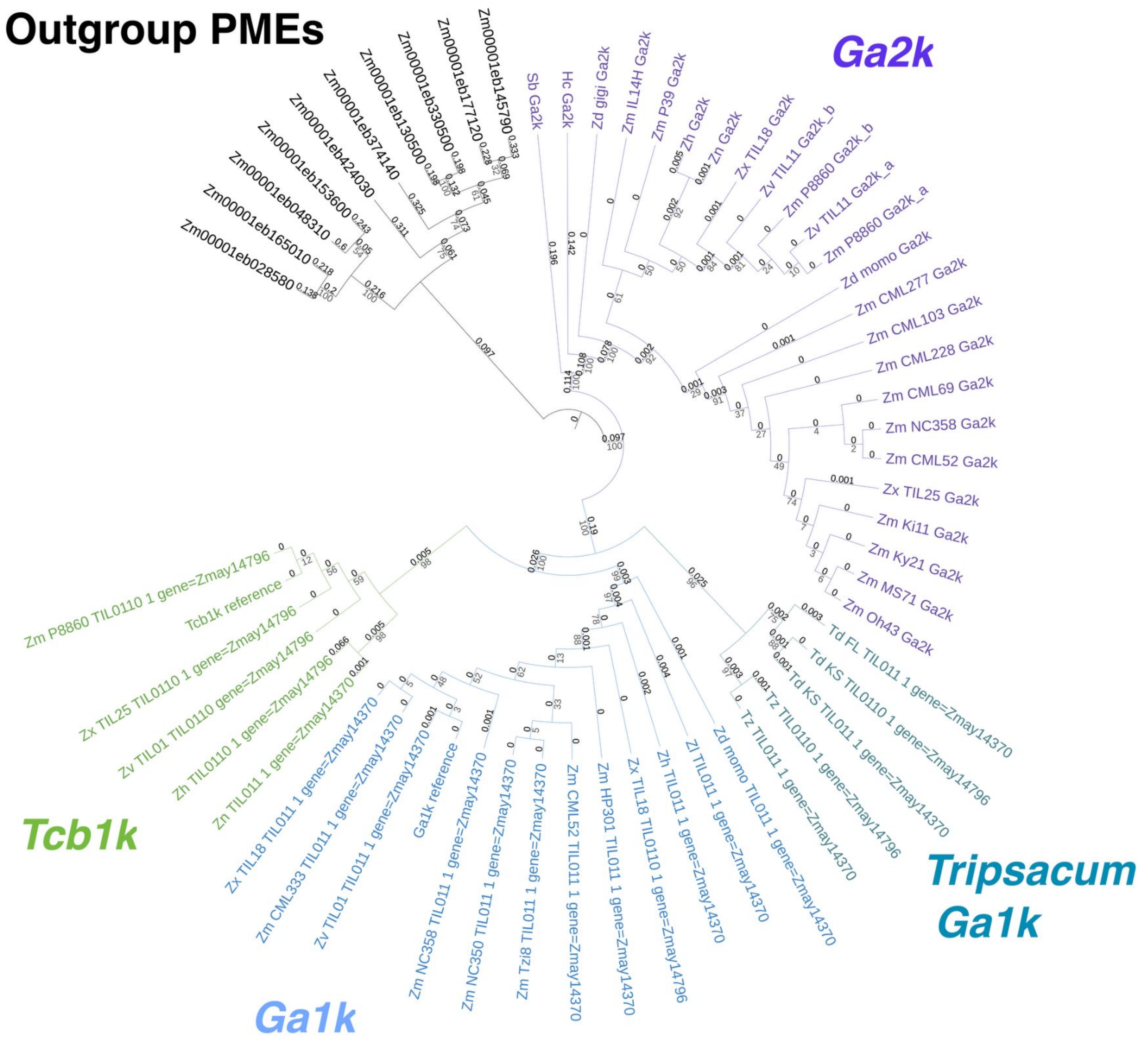

Supplemental Figure 2a
Putatively functional GA silk gene tree
Gene tree of GA silk gene sequences with CDS that can be translated into a full-length amino acid sequence with no premature stop codons. *Zea mays mays* PMEs as an outgroup. Tree is based on codon-aware nucleotide alignments of CDSs. Gene tree was built in RAxML and visualized in iTOL.

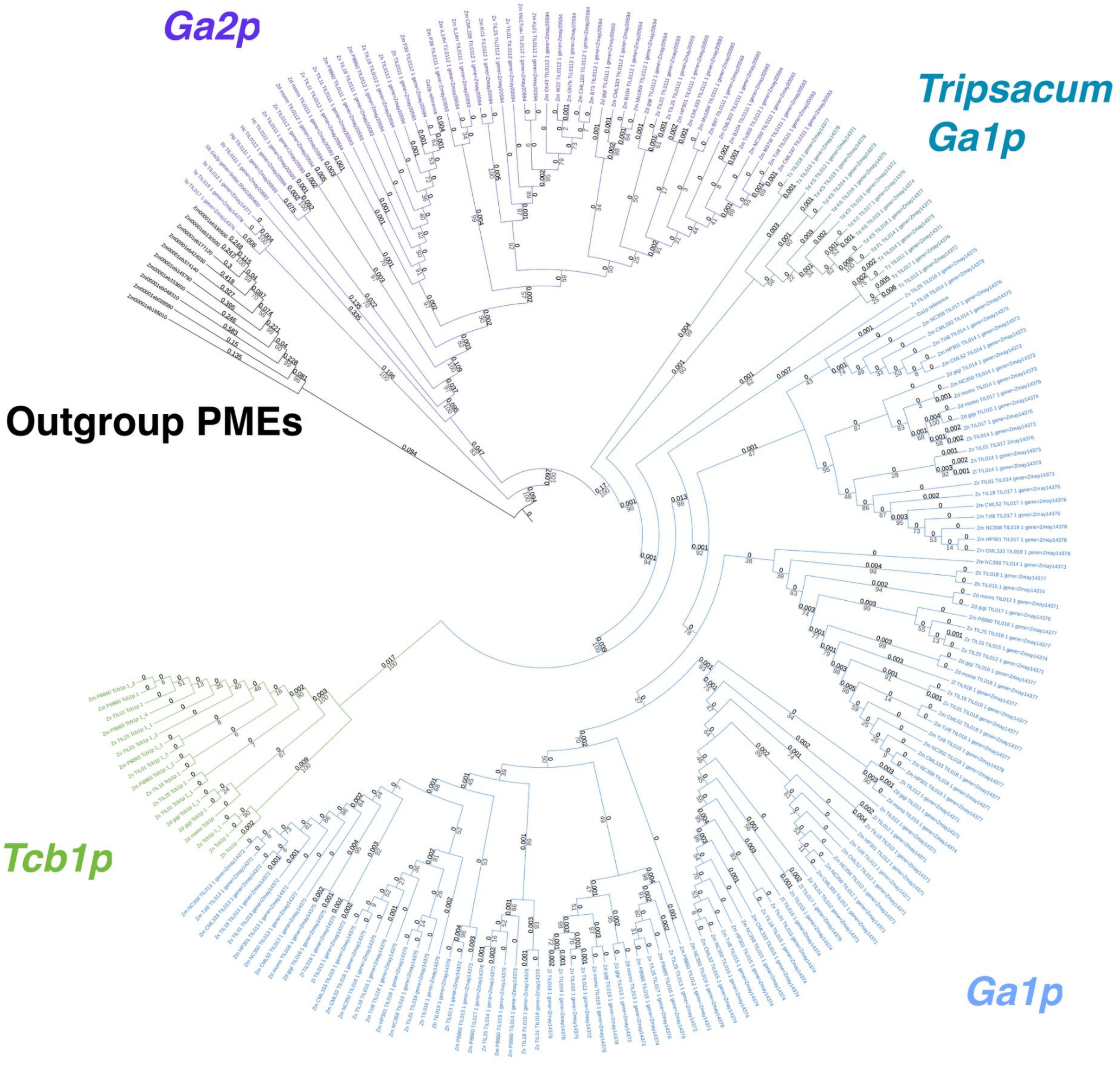

Supplemental Figure 2b
Putatively functional GA pollen gene tree
Gene tree of GA pollen gene sequences with CDS that can be translated into a full-length amino acid sequence with no premature stop codons. *Zea mays mays* PMEs as an outgroup. Tree is based on codon-aware nucleotide alignments of CDSs. Gene tree was built in RAxML and visualized in iTOL.

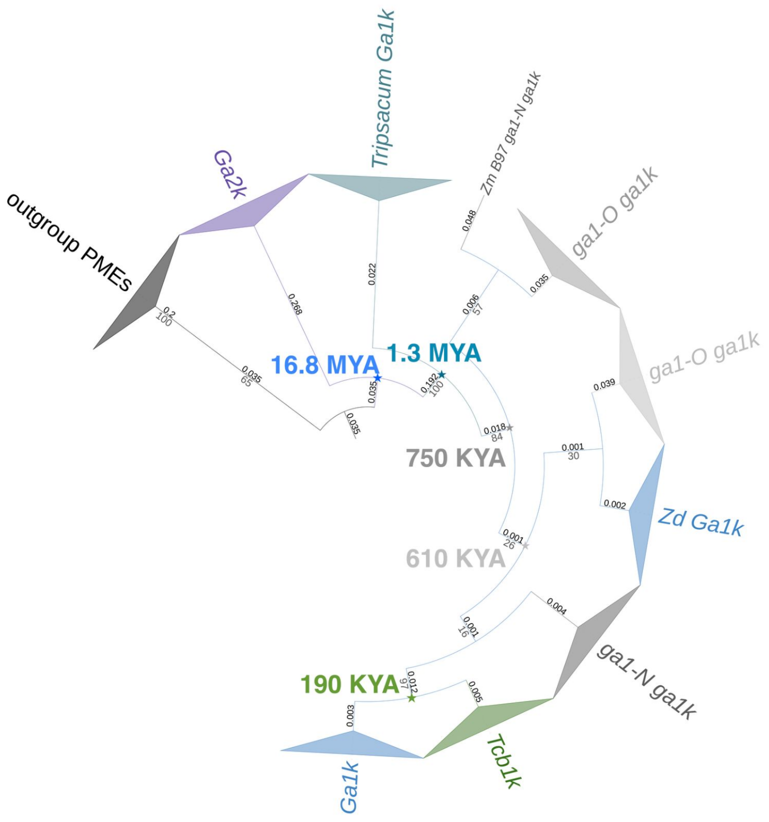

Supplemental Figure 3a:
GA silk estimated divergence times

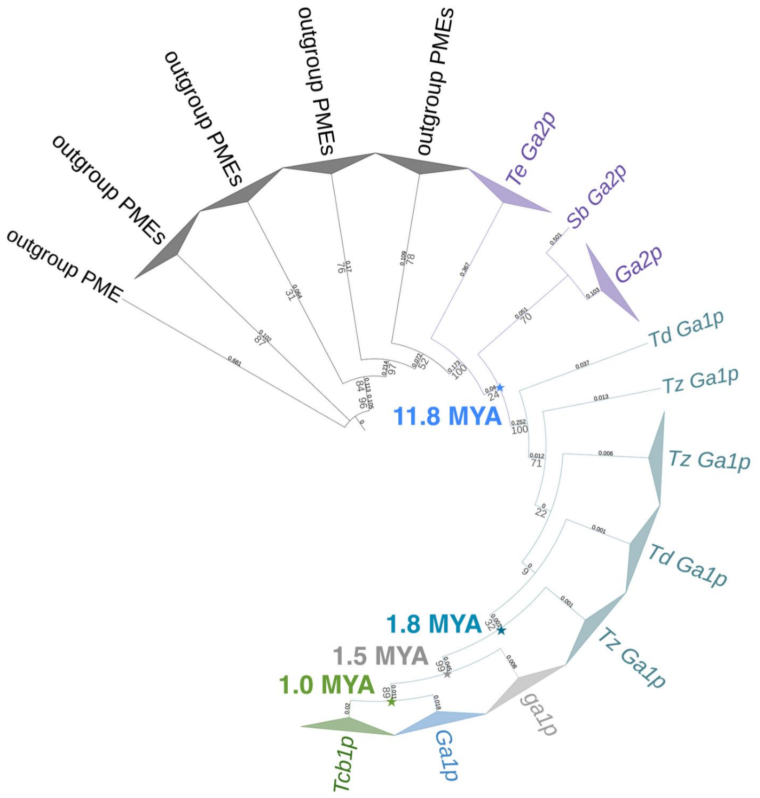

Supplemental Figure 3b:
GA pollen estimated divergence times

Supplemental Figure 3
Simplified GA gene trees with estimated divergence times

Gene trees simplified by locus with estimated ages. Ages were estimated via synonymous substitution rate and an assumed generation time of 1 year. *Zea mays mays* PMEs as an outgroup. Trees are based on codon-aware nucleotide alignments of CDSs. Gene trees were built in RAxML and visualized in iTOL.

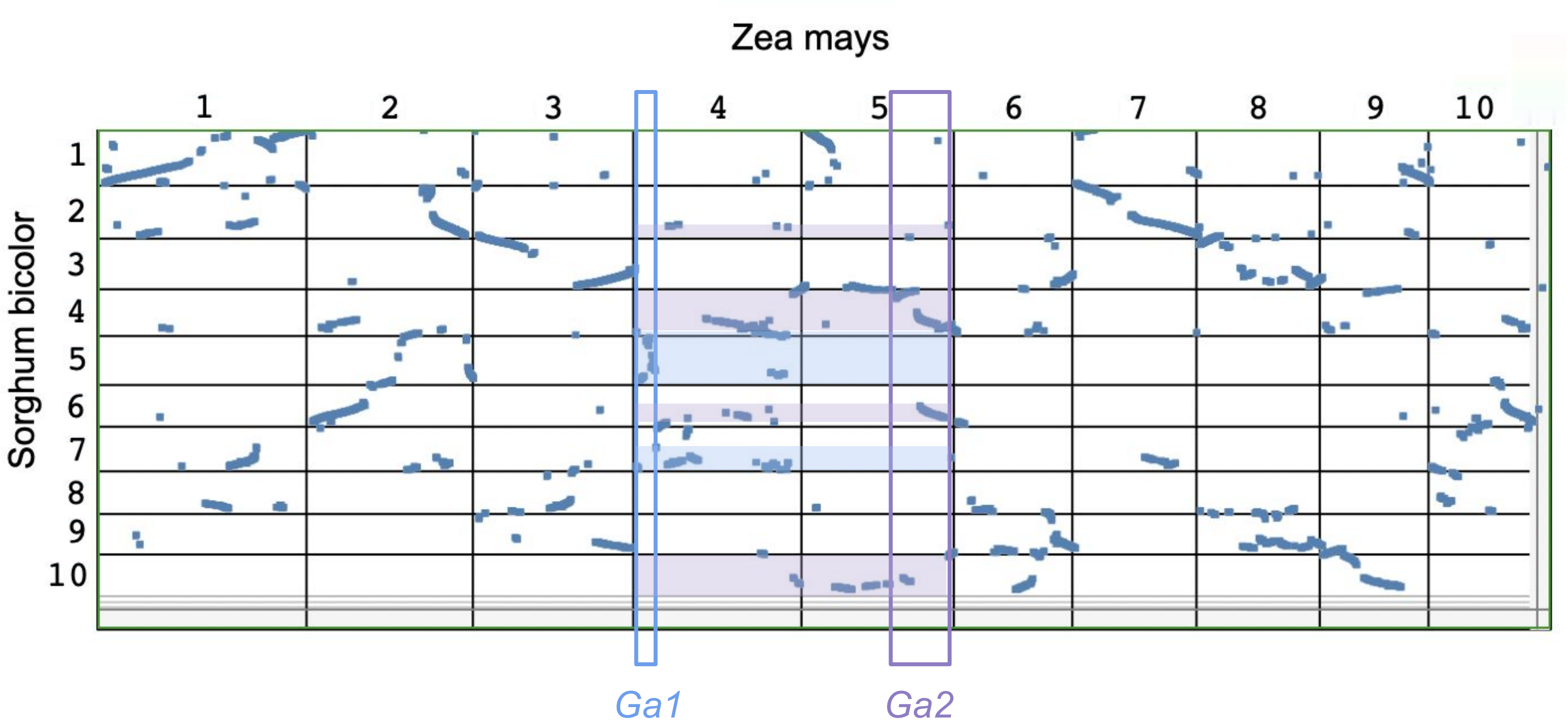

Supplemental Figure 4

***Ga2* and *Ga1* are not duplicates from the Tripsacinae WGD**

Whole genome synteny plot of orthologous genes between maize line B73 reference genome v4 (on the x-axis) and *Sorghum bicolor* reference genome v3.1.3 (on the y-axis) as an illustration of how synteny can where genomic regions are duplicates from a whole genome duplication (WGD). Sorghum and maize diverged before the Tripsacinae WGD, and fragments of the maize genome that arose during the WGD duplication are generally syntenic to the same region of the sorghum genome. For example, the end of maize chromosome 1 and beginning of maize chromosome 5 both map to the beginning of sorghum chromosome 1. The large regions of the maize genome flanking and containing *Ga1* (blue box) and *Ga2* (purple box) are not syntenic to the same region of the sorghum genome. Specifically, the *Ga1* region is syntenic mostly to sorghum chromosomes 5 and 7, which share no synteny at all with the *Ga2* region (as illustrated by blue highlighting). Reciprocally, the *Ga2* region shares synteny only with regions of the sorghum genome that are not syntenic to the *Ga1* region (as illustrated by purple highlighting). Synteny comparison and dotplot were generated in CoGe using SynMap (Haug-Baltzell et al. 2017): https://doi.org/10.1093/bioinformatics/btx144

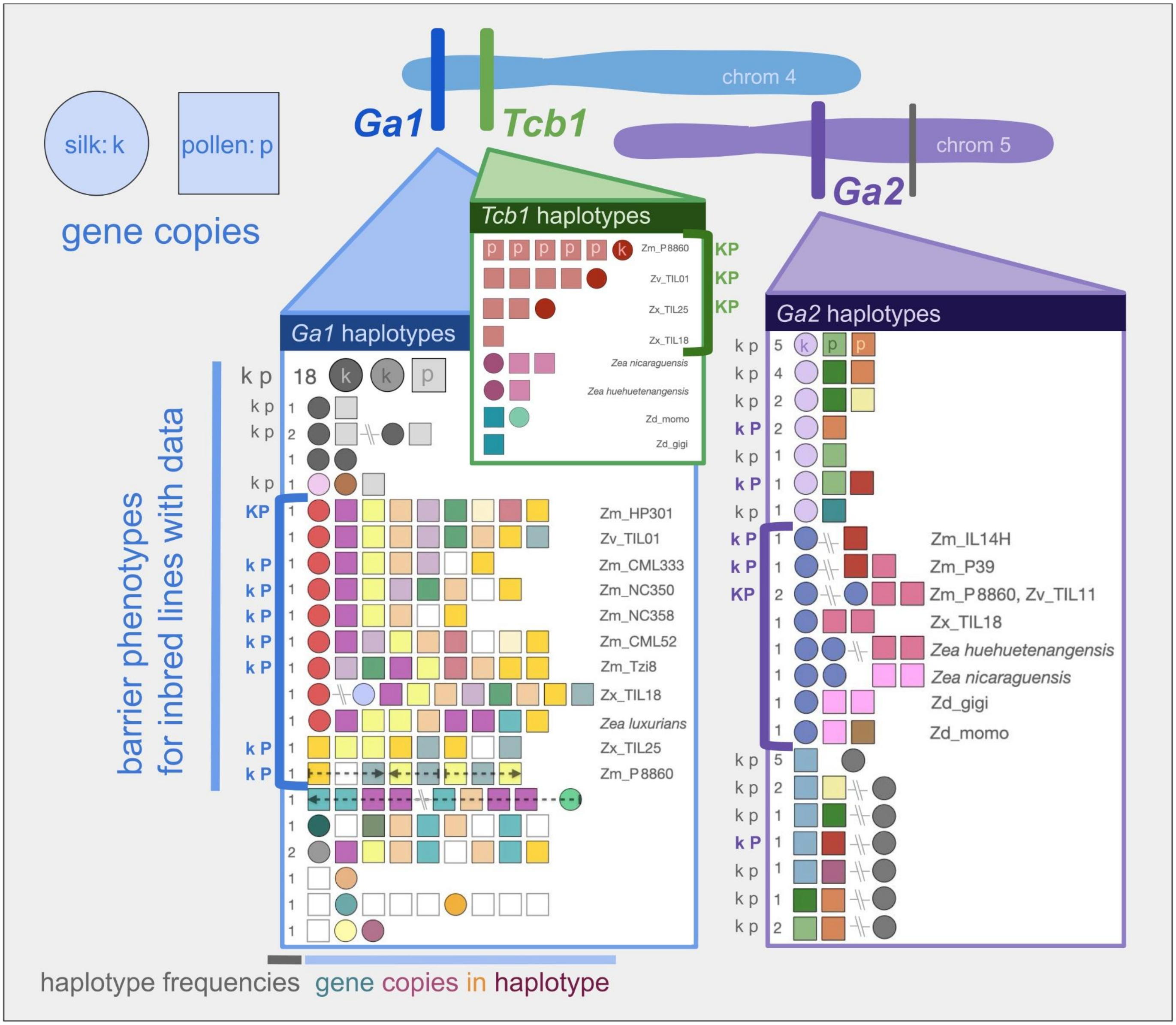

Supplemental Figure 5
Detailed haplotype diversity at each GA locus
Detailed view of haplotypes at the *Ga1* (blue) and *Tcb1* (green) loci on maize chromosome 4 and the *Ga2* (purple) locus on maize chromosome 5.
Each row of shapes represents a sequence of full-length gene copies on a haplotype from 5' to 3', where circles are silk genes and squares are pollen genes. Shared gene copy color represents shared gene copy sequence. Gene copies with stop codons are gray, and gene copies found in only one haplotype are white. Hashed lines between gene copies represent more than a kilobase of distance between copies. Location of the distal non-functional silk gene copy found in some *Ga2* haplotypes is also marked on chromosome 5 in gray. Gene copies on a haplotype are all in the same direction, except where indicated by arrows. Haplotype frequency is labeled in gray to the left of each haplotype, except for *Tcb1* haplotypes which are all singletons. Known barrier activity is marked for inbred lines phenotyped for both silk (k) and pollen (p) activity, where bolded, colored, capital letters represent an active barrier (K) or ability to overcome the barrier (P). Haplotypes which seem similar to fully active haplotypes (indicated by brackets) are labeled with species and line names. Condensed species labels for genotypes with line names are: Zm (*Z. mays mays*), Zv (*Z. mays parviglumis*), Zx (*Z. mays mexicana*), and Zd (*Z. diploperennis*).

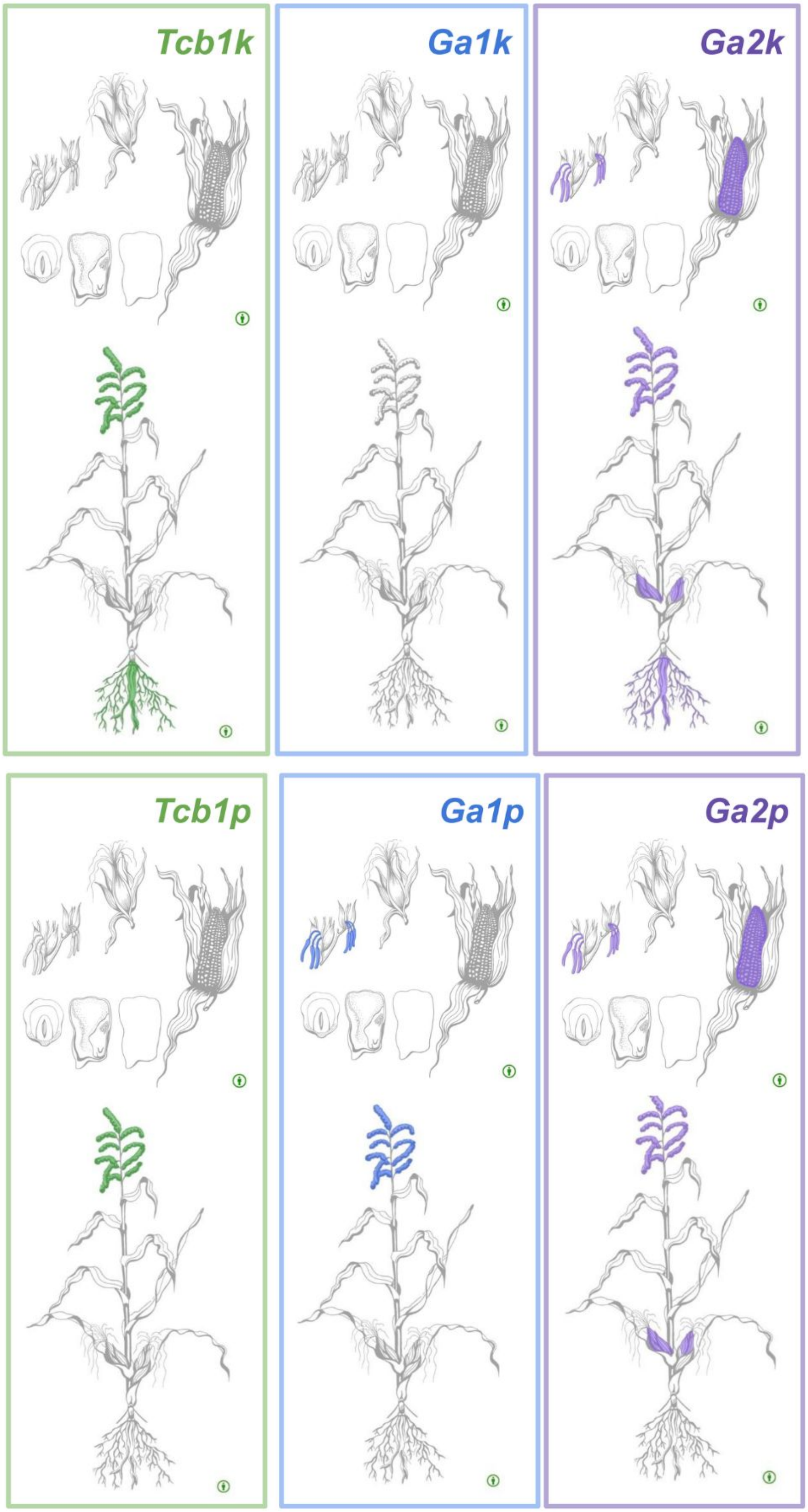

Supplemental Figure 6
Expression anatograms by gene
Tissues with RNA expression in at least one genome at >= 5 rpkm. See supplemental data for expression data sources. Anatogram templates are from Moreno et al 2022 https://doi.org/10.1093/nar/gkab1030.

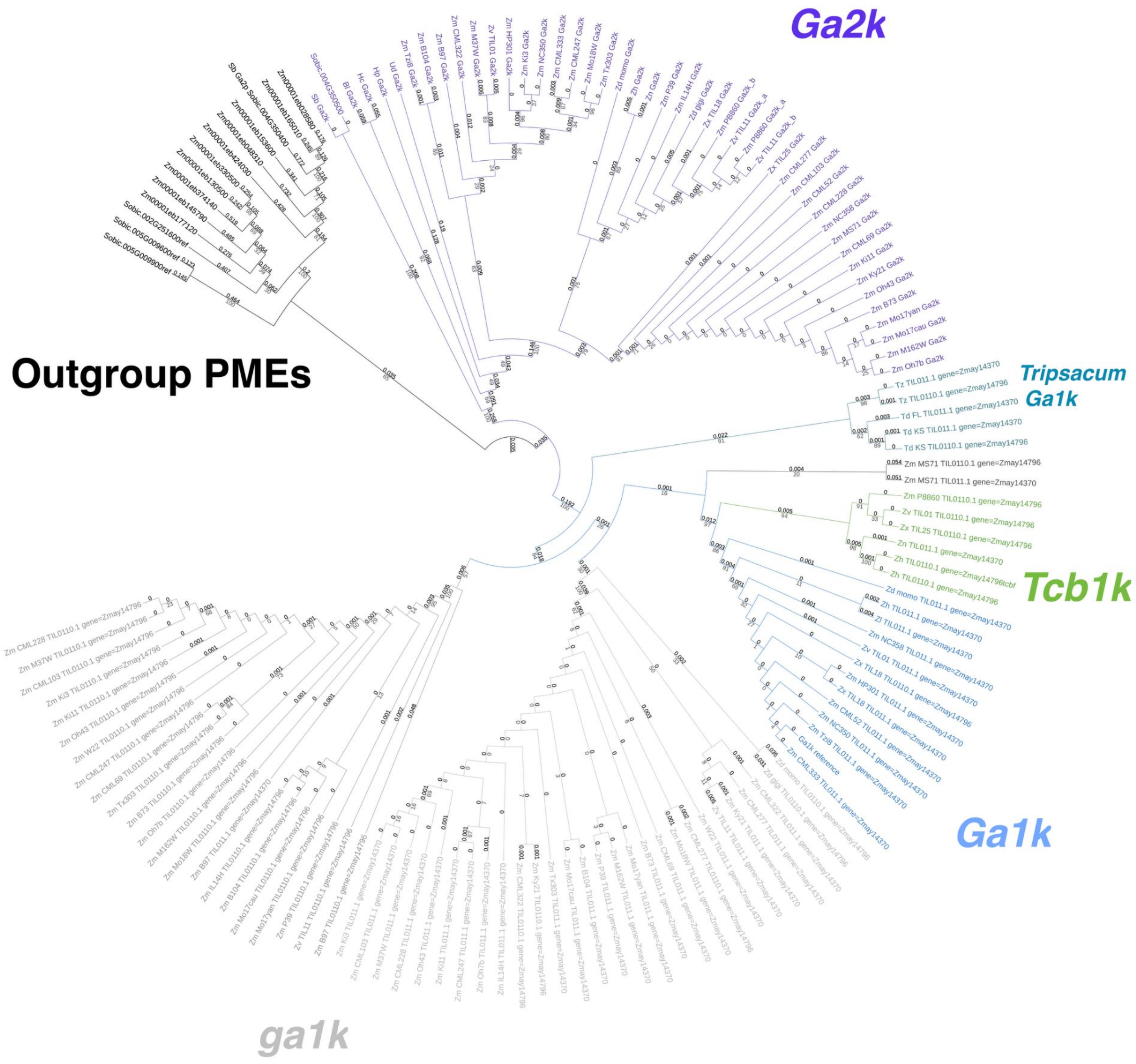

Supplemental Figure 7a
GA silk gene tree
Gene tree of all full-length GA silk gene sequences with *Zea mays mays* PMEs as an outgroup. Tree is based on nucleotide alignments of CDSs. Gene tree was built in RAxML and visualized in iTOL.

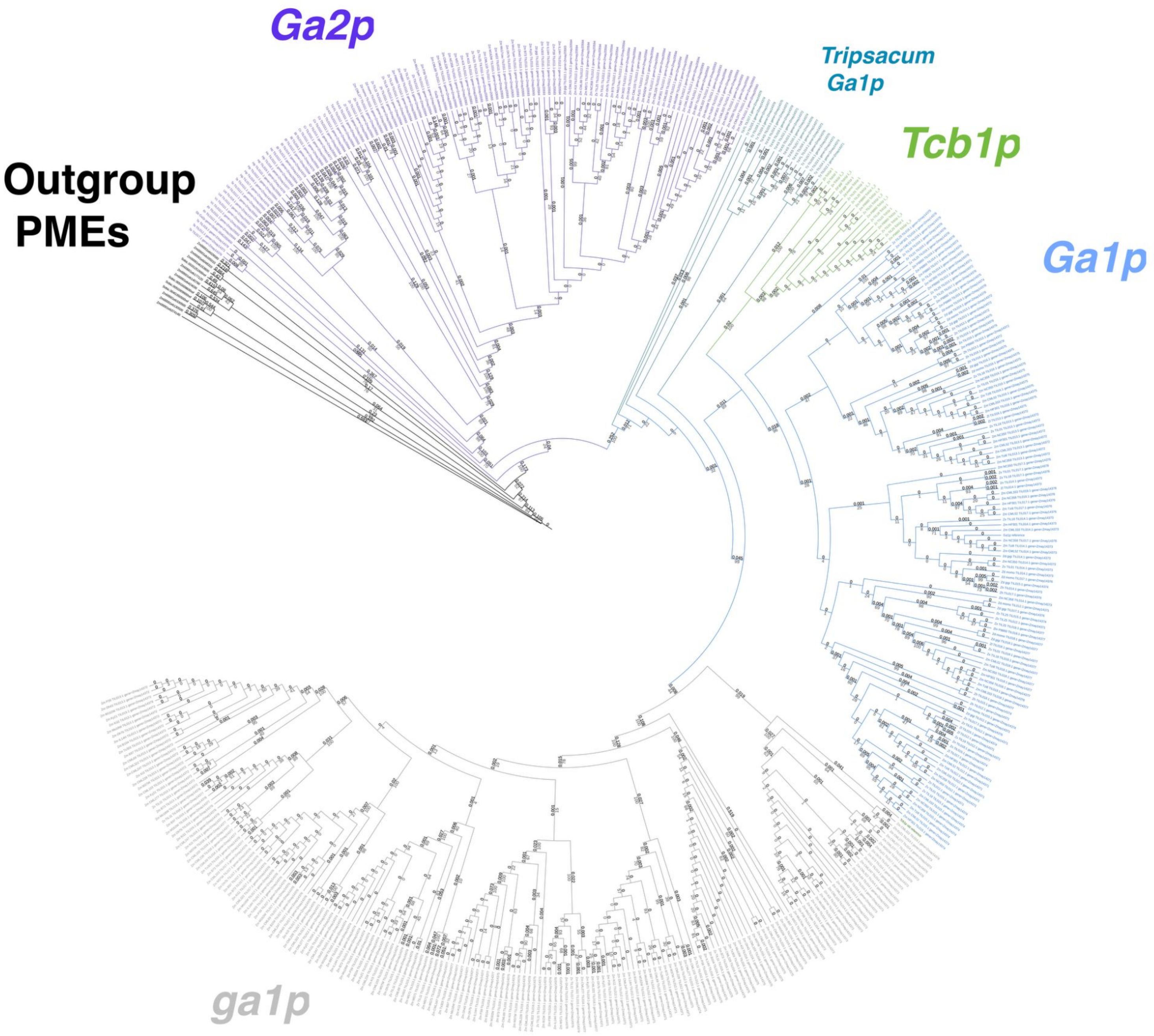

Supplemental Figure 7b
GA pollen gene tree
Gene tree of all full-length GA pollen gene sequences with *Zea mays mays* PMEs as an outgroup. Tree is based on nucleotide alignments of CDSs. Gene tree was built in RAxML and visualized in iTOL.

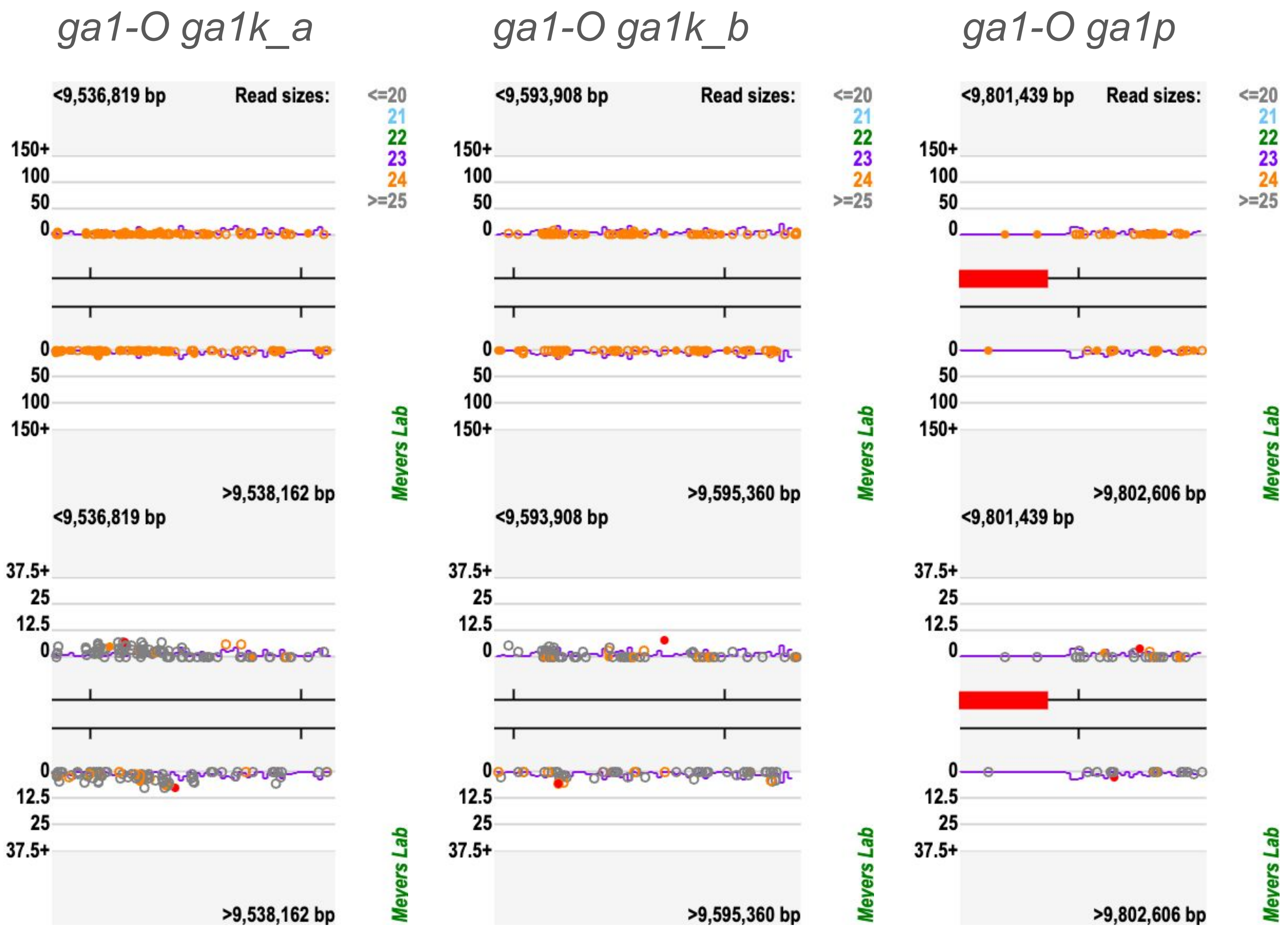

Supplemental Figure 8
24nt siRNA hits and phase scoring for each ga1-O 'gene' in B73

24nt siRNA hits (upper panels): Each dot represents a 24-nt siRNA hit, or regions in the B73 genome with exact sequence match to the siRNA. Hollow dots represent unique hits where the siRNA involved in the hit does not match the sequence of any other part of the B73 genome. Red bar on ga1p panel represents overlap with a B73 gene model for ZmPME3, which has been previously reported as the *Ga1* pollen factor. 24-nt siRNAs depicted are from the same lines and anther tissues described in main text. **Phasing Analysis (lower panels):** Each dot represents a "window" of ten cycles of small RNAs of length 24 nt, with the score for the degree of phasing indicated on the Y axis (scores calculated approximately as described by Howell et al., 2007). The red dot is the highest scoring window and has the best score in this region. Other colored dots are windows which are in phase with the highest scoring window -- exactly-in-phase windows appear as filled dots, and almost-in-phase (-1/+1) windows as hollow dots.

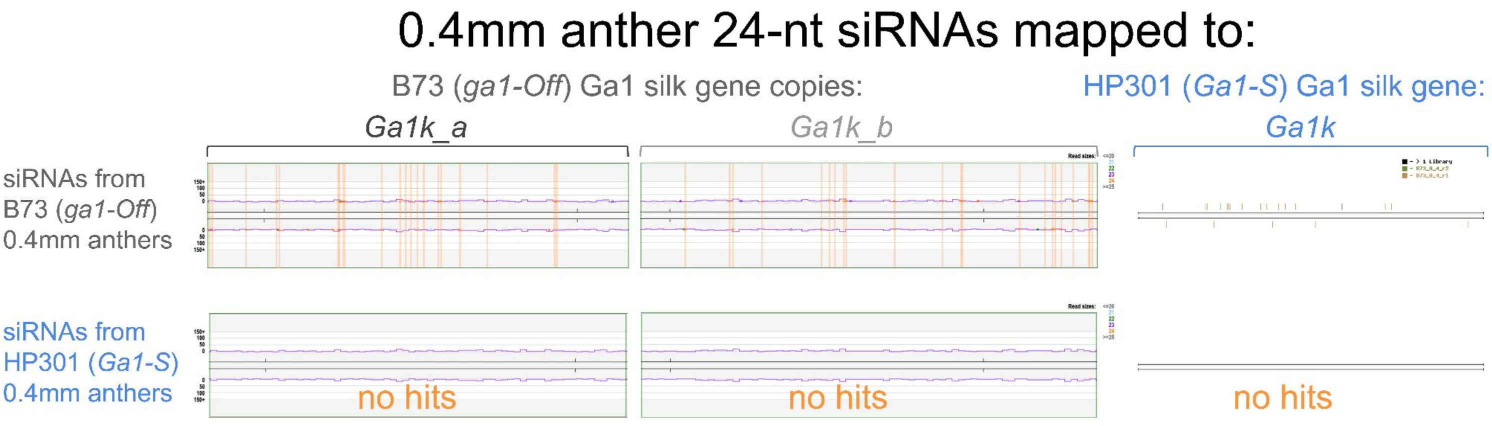

Supplemental Figure 9

0.4mm anther 24-nt siRNAs from *ga1-Off* line B73 map uniquely to the *ga1-Off Ga1k* gene copies of the B73 genome but also to the functional *Ga1k* gene sequence from *Ga1-S* line HP301

0.4mm anther B73 (*ga1-Off*) 24-nt siRNA hits mapped to the B73 and HP301 *Ga1k* gene copies (upper panels):
In the left and middle plots, each dot represents a 24-nt siRNA hit, or regions in the B73 genome with exact sequence match to an siRNA sequence found in two 0.4mm anther B73 siRNA libraries. Hollow dots represent unique hits where the siRNA involved in the hit does not match the sequence of any other part of the B73 genome. These same hits are also highlighted in orange.

In the right plot, each tick mark represents a hit where the siRNAs from the same B73 libraries map to the functional HP301 *Ga1k* sequence. Because the hits are mapped only to the *Ga1k* sequence.

0.4mm anther HP301 (*Ga1-S*) 24-nt siRNA hits mapped to the B73 and HP301 *Ga1k* gene copies (lower panels):
Like above, each dot or tick mark would represent an siRNA hit where siRNAs from two 0.4mm anther HP301 siRNA libraries map to *Ga1k* gene copies. No siRNAs found in these libraries map to the non-functional B73 *Ga1k* gene copy sequences or the functional HP301 *Ga1k* gene sequence.

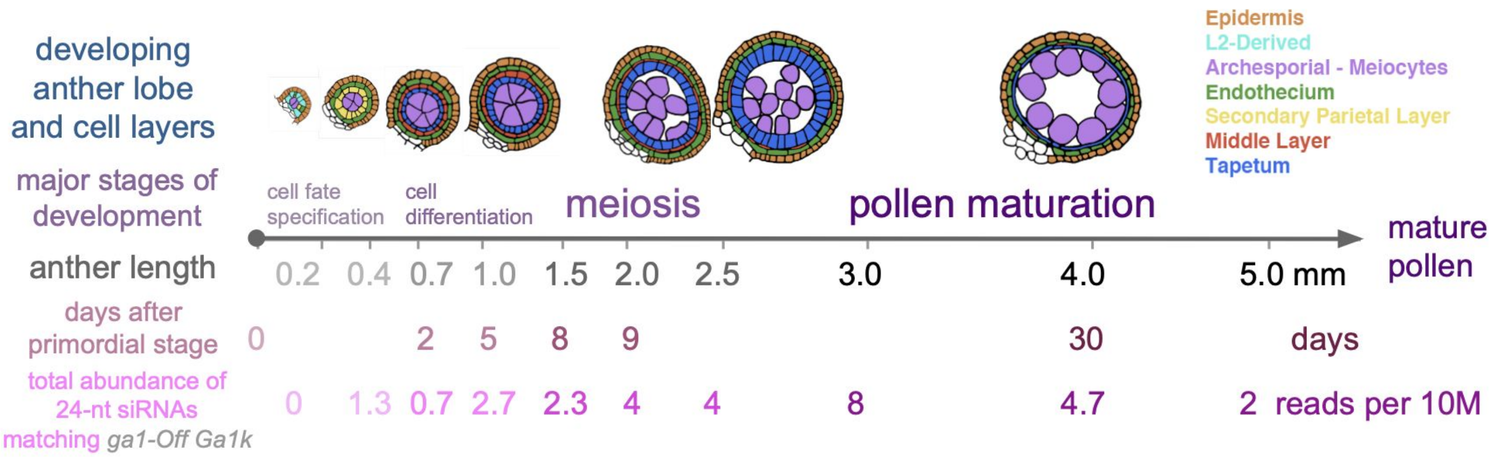

Supplemental figure 10
***ga1-Off*-associated 24-nt siRNAs are expressed across a month of anther development**
Figure adapted from Zhai et al. 2015:
https://doi.org/10.1073/pnas.1418918112

Anther developmental stages, spanning thirty days after the primordial stage, are diagrammed as a transverse cross-section of one of four lobes of the maize anther, with cell layers labeled by color (Zhai et al. 2015, Kelliher and Walbot 2011: https://doi.org/10.1016/j.ydbio.2010.11.005). Corresponding major stages of development (purple), anther length (gray), and days after primordial stage to reach anther lengths in B73 (maroon) are also labeled (Marchant and Walbot 2022: https://doi.org/10.1093/plcell/koac287, Zhai et al. 2015, Kelliher and Walbot 2011). In siRNA libraries of W23 [*ga1-Off*, see Supplemental figure 9] anthers at each labeled length, we searched for siRNAs that aligned to either or both of the two full-length copies of the non-coding *Ga1* silk gene models found in the conserved *ga1-Off* haplotype (Zhai et al. 2015, Nakano et al. 2020). For only the aligned 24-nt siRNAs, we summed the total abundance of siRNAs measured in reads per 10 million and average across 2-3 replicates per anther length (fuschia) (Zhai et al. 2015, Nakano et al. 2020).

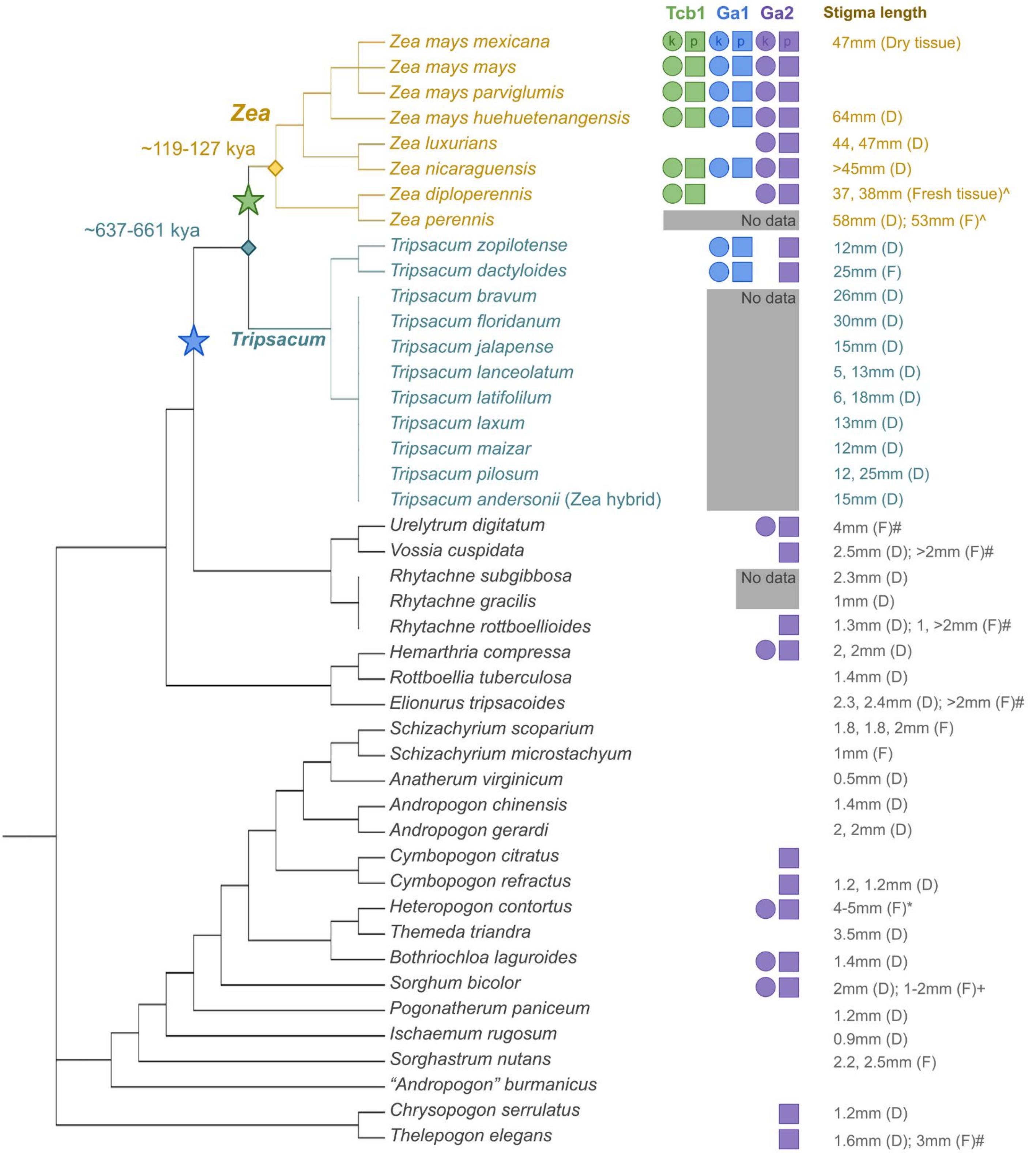

All stigma lengths measured by Elizabeth A Kellogg from fresh plant tissue or dried herbaria samples [see supplemental data]
Unless otherwise noted: * (Drisya and Pradeep 2020), + (Takanashi et al 2021),
(Stapf 1917 Flora of Tropical Africa Vol IX Part 1 Ed. Sir David Prain), ^ (measured by Jeffrey Ross-Ibarra from fresh tissue)

Supplemental Figure 11
Stigma length and GA loci PAV on Andropogoneae species tree
Tree of all species we searched for GA loci gene sequences with stigma (silk) length and GA loci gene presence. For more information on GA PAV, species tree, and divergence times, see main text.

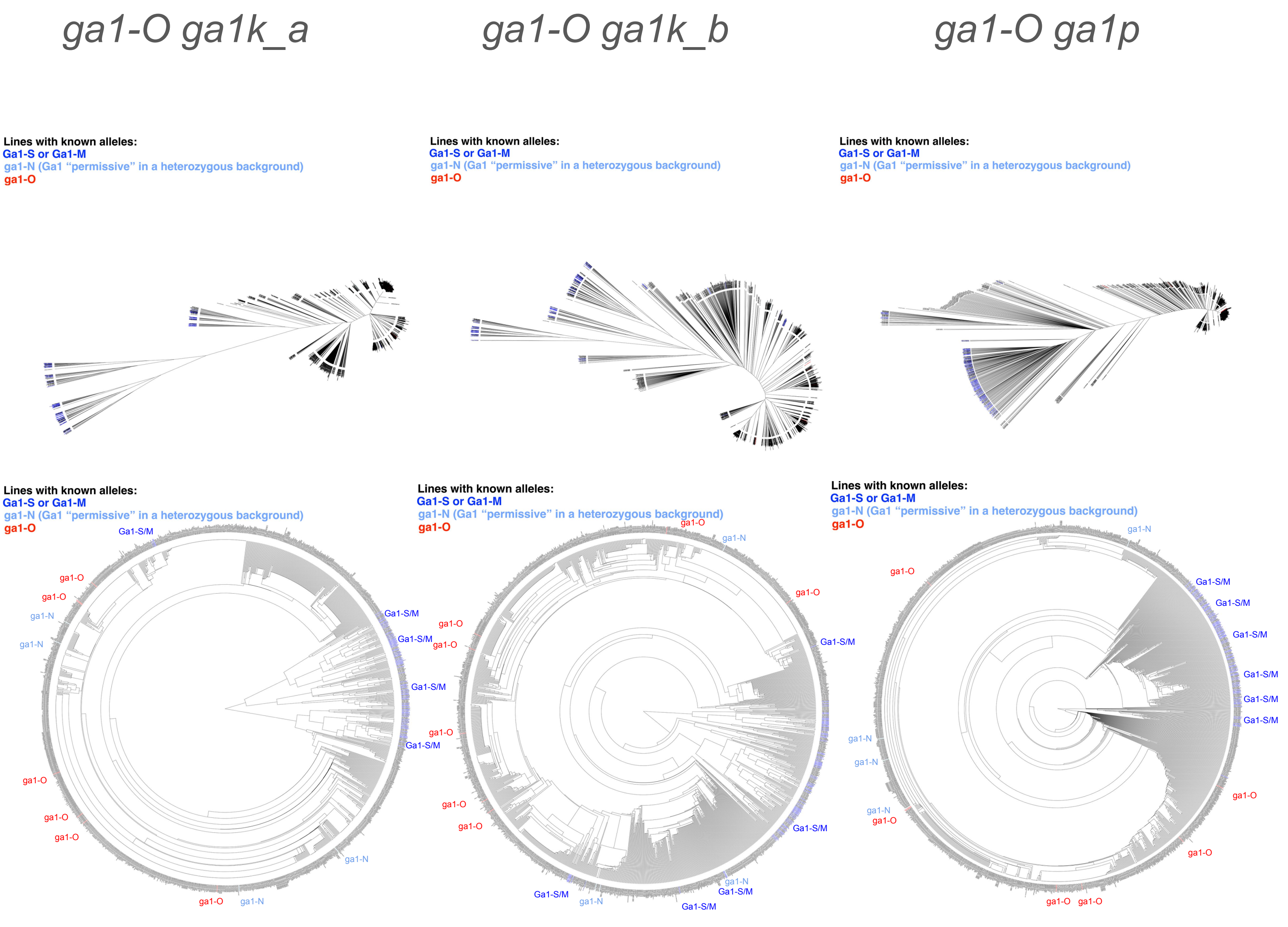

Supplemental Figure 12
SNP trees for GA loci across diverse maize lines
Neighbor-joining trees of SNPs in diverse maize lines alignment to the B73 *ga1k* and *ga1p* gene regions in the B73 *ga1-O* allele. Tips representing lines for which Ga1 barrier phenotypes are known are colored by allele type. Clades including lines with known phenotypes are additionally annotated with the allele status. While *ga1-O* and *Ga1-S* or *Ga1-M* lines are not found in similar parts of the SNP trees, *ga1-N* lines are sometimes grouped with *ga1-O* and sometimes grouped with *Ga1* lines, as expected for a phenotype that is the result of deleterious mutations and could therefore have easily arisen multiple times. Aligned SNPs were accessed through HapMap 4 on the Maize Genetics DataBase (MaizeGDB) website, which we also used to build the NJ trees. HapMap 4 and MaizeGDB: Andorf et al 2024: https://doi.org/10.1101/2024.04.30.591904; Hufford et al 2021: doi:10.1126/science.abg5289; Grzybowski et al 2023: doi:10.1111/tpj.16123; Woodhouse et al 2021: doi:10.1186/s12870-021-03173-5
