# Supplementary material for: Molecular evolution of a reproductive barrier in maize and related species
Source: Genetics. 2025 May 8;230(3):iyaf085. doi: 10.1093/genetics/iyaf085 (PMC12239200; doi:10.1093/genetics/iyaf085)
Supplement: iyaf085_Supplementary_Data [file iyaf085_supplementary_data.zip › Supplementary Literature Cited_iyaf085.docx]

**Supplementary Literature Cited**

**Molecular evolution of a reproductive barrier in maize and related species**

**E. Cryan et al.,** <https://doi.org/10.1093/genetics/iyaf085>

**Andorf CM, Ross-Ibarra J, Seetharam AS, Hufford MB, Woodhouse MR. 2025.** A unified VCF dataset from nearly 1,500 diverse maize accessions and resources to explore the genomic landscape of maize. *G3 Genes|Genomes|Genetics* . 15(2). <https://doi.org/10.1093/g3journal/jkae281>.

**Drisya V, Pradeep AK.** 2020. On reinstating Heteropogon Allionii (Poaceae: Panicoideae). *Phytotaxa* . 429(2):157–166. doi:10.11646/phytotaxa.429.2.6.

**Grzybowski MW, Mural RV, Xu G, Turkus J, Yang J, Schnable JC.** 2023. A common resequencing-based genetic marker data set for global maize diversity. *Plant J.* 113(6):1109–1121. doi:10.1111/tpj.16123.

**Haug-Baltzell A, Stephens SA, Davey S, Scheidegger CE, Lyons E. 2017.** SynMap2 and SynMap3D: web-based whole-genome synteny browsers. *Bioinformatics* (Oxford, England). 33(14):2197–2198. doi:10.1093/bioinformatics/btx144.

**Takanashi H, Shichijo M, Sakamoto L, Kajiya-Kanegae H, Iwata H, Sakamoto W, Tsutsumi N. 2021.** Genetic dissection of QTLs associated with spikelet-related traits and grain size in Sorghum. *Sci Rep*. 11(1):9398. doi:10.1038/s41598- 021-88917-x.

**Waterhouse AM, Procter JB, Martin DMA, Clamp M, Barton GJ.** 2009. Jalview version 2–a multiple sequence alignment editor and analysis workbench. *Bioinformatics* (Oxford, England). 25(9):1189–1191. doi:10.1093/bioinformatics/btp033.
